# Lactate promotes neuronal differentiation of SH-SY5Y cells by lactate-responsive gene sets through NDRG3-dependent and -independent manners

**DOI:** 10.1016/j.jbc.2023.104802

**Published:** 2023-05-10

**Authors:** Yidan Xu, Joji Kusuyama, Shion Osana, Satayuki Matsuhashi, Longfei Li, Hiroaki Takada, Hitoshi Inada, Ryoichi Nagatomi

**Affiliations:** 1 Department of Medicine and Science in Sports and Exercise, Tohoku University Graduate School of Medicine, Sendai, Japan; 2 Frontier Research Institute for Interdisciplinary Sciences, Tohoku University, Sendai, Japan; 3 Division of Biomedical Engineering for Health and Welfare, Tohoku University Graduate School of Biomedical Engineering, Sendai, Japan; 4 Department of Biosignals and Inheritance, Graduate School of Medical and Dental Sciences, Tokyo Medical and Dental University (TMDU), Tokyo, Japan; 5 Graduate School of Informatics and Engineering, University of Electro-Communications, Tokyo, Japan; 6 Department of Developmental Neuroscience, Tohoku University Graduate School of Medicine, Sendai, Japan

**Keywords:** neurogenesis, NF-H, TUBB3, NSE, MAP2, ID2, RUNX1T1, GPR81, SYT4

## Abstract

Lactate serves as the major glucose alternative to an energy substrate in the brain. Lactate level is increased in the fetal brain from the middle stage of gestation, indicating the involvement of lactate in brain development and neuronal differentiation. Recent reports show that lactate functions as a signaling molecule to regulate gene expression and protein stability. However, the roles of lactate signaling in neuronal cells remain unknown. Here, we showed that lactate promotes the all stages of neuronal differentiation of SH-SY5Y and Neuro2A, human and mouse neuroblastoma cell lines, characterized by increased neuronal marker expression and the rates of neurites extension. Transcriptomics revealed many lactate-responsive genes sets such as *SPARCL1* in SH-SY5Y, Neuro2A, and primary embryonic mouse neuronal cells. The effects of lactate on neuronal function were mainly mediated through monocarboxylate transporters 1 (MCT1). We found that NDRG family member 3 (NDRG3), a lactate-binding protein, was highly expressed and stabilized by lactate treatment during neuronal differentiation. Combinative RNA-seq of SH-SY5Y with lactate treatment and NDRG3 knockdown shows that the promotive effects of lactate on neural differentiation are regulated through NDRG3-dependent and independent manners. Moreover, we identified TEA domain family member 1 (TEAD1) and ETS-related transcription factor 4 (ELF4) are the specific transcription factors that are regulated by both lactate and NDRG3 in neuronal differentiation. TEAD1 and ELF4 differently affect the expression of neuronal marker genes in SH-SY5Y cells. These results highlight the biological roles of extracellular and intracellular lactate as a critical signaling molecule that modifies neuronal differentiation.

The biological significance of lactate has long been confined as a metabolite derived from glycolysis (1). Lactate is generated from exercise and the contraction of skeletal muscles through anaerobic glycolysis, and excess lactate is oxidized by the liver, heart, kidney, and brain (2). Most types of cells consume glucose as the main energy source; however, neurons predominantly metabolize glucose in the pentose phosphate pathway to produce reduced equivalents in the form of NADPH (3). Alternatively, neurons preferably consume lactate and lactate-derived pyruvate as their mitochondrial energy substrate. Astrocyte-neuron lactate shuttle model, a lactate transportation system from astrocyte to neuron *via* transmembrane monocarboxylate transporters (MCTs), explains that fundamental neuronal activity is maintained by glycogenolysis- and glycolysis-induced lactate production in astrocytes (4). The identification of lactate as an energy substrate for neuronal cells has scientific attention to the more active roles of lactate in the central nervous system (5, 6). For example, lactate import is necessary for the maintenance of long-term potentiation and elicited synaptic strength (7), neuronal excitability (8), and neurite outgrowth (9). Lactate promotes the proliferation and mitochondrial length in radial glial progenitor cells (10), the release of norepinephrine in coeruleus neuron (11), and learning memory in the hippocampus (12). Notably, lactate is accumulated in the brain during the gestational stage (13), indicating the potential roles of lactate for brain development and neuronal differentiation. Nevertheless, the mechanism by which lactate mediates the functional alteration in the neuronal system has not been fully elucidated.

Recent studies showed that lactate has dual roles as an energy substrate and a cellular signaling molecule in several types of cells. Intracellular and extracellular lactate levels regulate a variety of gene expressions in muscle cells (14), macrophages (15), vasculogenic stem cells (16), and neurons (17). Lactate stimulation directly activates ERK phosphorylation (17) and JAK2-STAT5 signaling pathways (18). Furthermore, incorporated extracellular lactate induces target protein modification as lactylation and changes anti-tumor function in Treg cells (19). Given the growing evidence that lactate provides signal-regulatory functions in various cell types under physiological and pathological conditions, we hypothesized that lactate affects neuronal function through changes in signaling patterns and comprehensive gene expression.

SH-SY5Y, a human neuroblastoma cell line derived from a metastatic bone tumor from a 4-year-old patient, is a well-accepted and commonly used *in vitro* model to explore neuronal cell differentiation. SH-SY5Y cells have a stable karyotype consisting of 47 chromosomes and can be differentiated from a neuroblast-like state into specific neuronal sub-types (20) and mature neurons (21) showing representative features of neuronal differentiation. For example, neurite outgrowth, which is essential in the formation of nerve connection and brain development (22), can be easily evaluated by SH-SY5Y differentiation model. Since the errors in the neurite outgrowth process cause deficiencies in axonal wiring, synapse deformation, and brain maturation inducing neurodevelopmental disorders such as autism (23), the early phase of neuronal differentiation is an essential step for fundamental brain functions.

Here, we show that lactate stimulation promotes neuronal differentiation of SH-SY5Y cells. Transcriptome analysis revealed the representative sets of lactate-responsive genes and lactate-regulated pathways. NDRG family member 3 (NDRG3), a lactate-binding protein, is one of the most important upstream mediators to enhance neuronal differentiation. The combinative RNA-seq of NDRG3 knock downed- and lactate treated-SH-SY5Y cells revealed that TEA domain family member 1 (TEAD1) and E74 like ETS transcription factor 4 (ELF4) are co-regulatory transcription factors in lactate-NDRG3 signaling axis. Our findings show that lactate plays a critical role in neuronal differentiation and multiple lactate-induced signaling pathways modify neuronal functions.

## Results

### Lactate treatment promotes the neural differentiation of neuroblastoma cell lines

**Figure 1:**
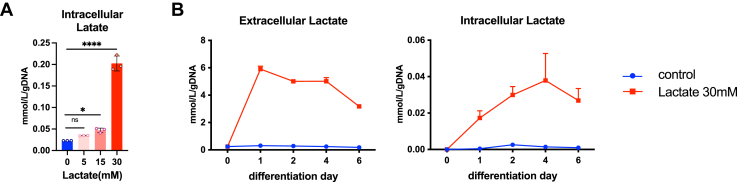
**Exogenous lactate stimulation affects extracellular and intercellular lactate concentration in SH-SY5Y cells.***A*, intracellular lactate levels in 5, 15, and 30 mM lactate treated- SH-SY5Y (n = 3). All data are reported as means ± SEM. ∗*p* < 0.05, ∗∗*p* < 0.01, ∗∗∗*p* < 0.001, ∗∗∗∗*p* < 0.0001, *versus* control. Statistical significance was determined by one-way ANOVA with Dunnett tests. *B*, extracellular and intracellular lactate levels during SH-SY5Y differentiation under 30 mM lactate treatment (n = 3).

To examine the effects of lactate on neural cell differentiation, we first treated SH-SY5Y, a human neuroblastoma cell line with 0, 5, 15, or 30 mM lactate in the differentiation media for 1 day and analyzed the levels of intracellular lactate. The intracellular concentration of lactate was markedly increased only in 30 mM lactate treatment compared to 5 and 15 mM (Fig. 1*A*). We also examine the effects of daily 30 mM lactate treatment on extracellular and intracellular lactate concentration during the differentiation process. Consistently, the intercellular concentration of lactate was augmented from day 1 and kept at three- to six-fold increases during neural differentiation by 30 mM lactate treatment (Fig. 1*B*). Therefore, we selected 30 mM lactate as the optimal concentration for further experiments.

**Figure 2:**
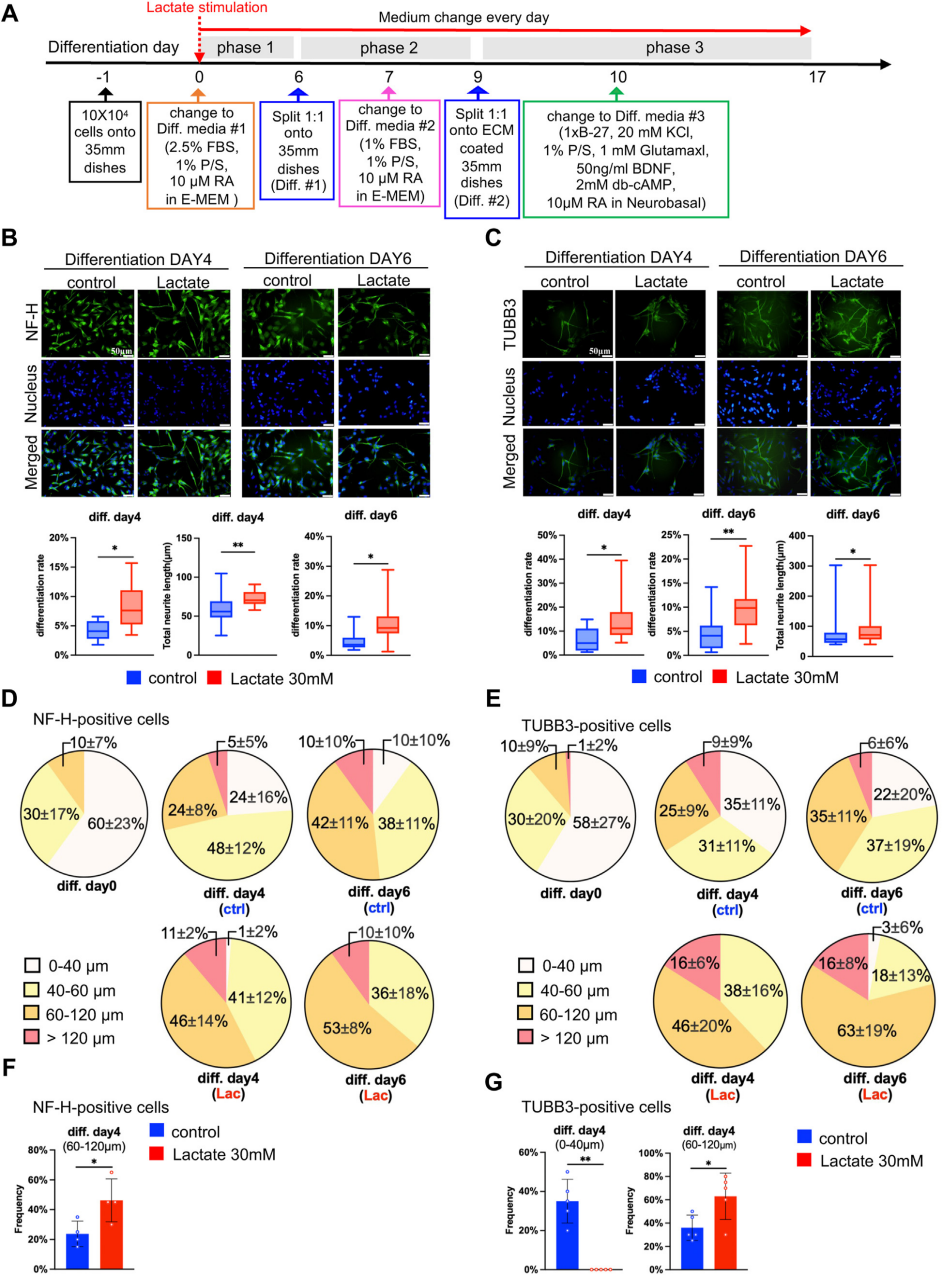
**Lactate promotes neurite outgrowth and differentiation rates of SH-SY5Y cells.***A*, timetable and reagent recipes of SH-SY5Y differentiation. *B* and *C*, representative immunocytochemistry images of differentiated SH-SY5Y with or without 30 mM lactate treatment. Cells were stained with NF-H (*B*) or TUBB3 (*C*). Scale bar = 50 μm. The differentiation rate (% of cells that bore at least one neurite longer than 40 μm) and the average length of total neurites (all neurites per differentiated cell) of differentiation on day 4, and day 6 were quantified. Neurite length is indicated as average neurite length ± standard error. At least 55 cells/group were analyzed from each experiment (n = 12), and values were presented as mean ± SEM. ∗*p* < 0.05, ∗∗*p* < 0.01, *versus* control, Statistical significance was determined by unpaired two-tailed *t* test. *D* and *E*, the percentage of neurite length of 0 to 40 μm, 40 to 60 μm, 60 to 120 μm, >120 μm at differentiation day 0, day 4, day 6 in NF-H- (*D*) and TUBB3 (*E*) -positive cells. The total differentiated cell number at differentiation day 0, day 4, and day 6 were calculated as 1, respectively. *F* and *G*, the % of neurite length of 0 to 40 μm, 60 to 120 μm at differentiation day 4 in NF-H- (*F*) and TUBB3 (*G*) -positive cells, respectively. At least 21 cells/group were analyzed from each experiment (n = 4 or 5), and values were presented as mean ± SEM. ∗*p* < 0.05, ∗∗*p* < 0.01, *versus* control. Statistical significance was determined by unpaired two-tailed *t* test.

We next induced the neural differentiation of SH-SY5Y cells following three-step protocol (Fig. 2*A*) in the addition of 30 mM lactate. SH-SY5Y cells were immunohistochemically stained with neurofilament-H (NF-H) (Fig. 2*B*) and beta III Tubulin (TUBB3) (Fig. 2*C*), the specific neuron markers expressed in cell body, neurites, and axon, at days 4 and 6. Lactate treatment promoted the cell differentiation rate (% of cells that bore at least one neurite longer than 40 μm) at days 4 and 6 and the total neurite length (average length of all neurites per differentiated cell) at day 6 in NF-H-positive cells. Similarly, TUBB3-positive cells have increased at days 4 and 6 and the prolonged neurites at day 6. Additionally, since the median of neurites length was approximately 60 μm (Fig. S1, *A* and *B*), we categorized all differentiated SH-SY5Y cells into four groups with neurite lengths of 0 to 40, 40 to 60, 60 to 120 (2-fold of median), and >120 μm and quantified the percentage of each group to analyze the elongation of neurite length during the differentiation under lactate treatment. Lactate treatment increased the percentages of 60 to 120 μm neurites on differentiation day 4 in both NF-H- (Fig. 2, *D* and *F*) and TUBB3- (Fig. 2, *E* and *G*) positive cells. Additionally, lactate treatment suppressed the percentages of 0 to 40 μm neurites on differentiation day 4 in TUBB3- (Fig. 2*G*) positive cells. These results indicate that lactate treatment promotes neural differentiation and the rates of neurites extension in SH-SY5Y cells.

**Figure 3:**
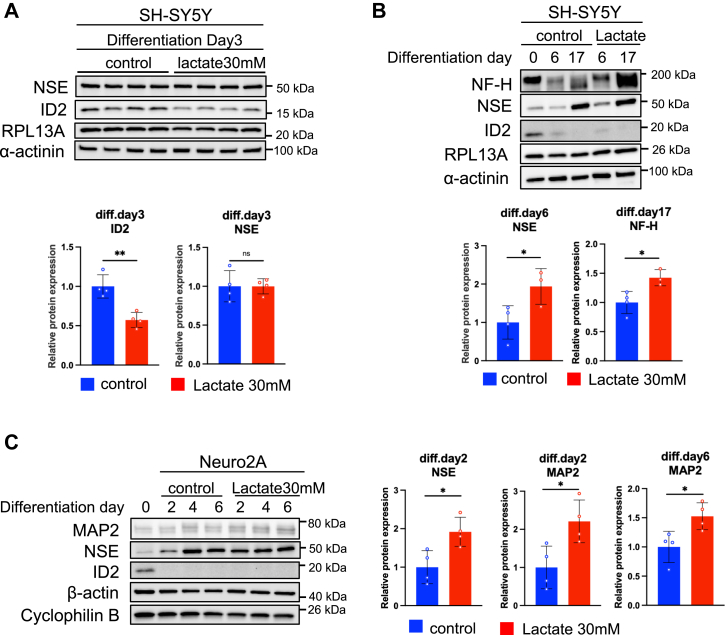
**Lactate promotes the protein expression of neuronal differentiation makers in SH-SY5Y cells and Neuro2A cells.***A*, protein level of NSE and ID2 at differentiation day 3 with or without 30 mM lactate supplementation in SH-SY5Y culture. *B*, protein level of NF-H, NSE, and ID2 during differentiation with or without 30 mM lactate supplementation in SH-SY5Y at day 0, 6, and 17. *C*, protein level of MAP2, NSE, and ID2 of the differentiation progress with or without 30 mM lactate supplementation in Neuro2A culture. Relative protein levels were normalized to the levels of RPL13A and α-actinin in SH-SY5Y and Cyclophilin B and β-actin in Neuro2A in the same samples and presented as fold change to control. All values were presented as mean ± SEM. ∗*p* < 0.05, ∗∗*p* < 0.01, *versus* control. Statistical significance was determined by an unpaired two-tailed *t* test.

To further determine the effects of lactate on all three phases of neuronal differentiation in SH-SY5Y cells (Fig. 2*A*) (21), we examined the protein expression of neuron-specific enolase (NSE) (24, 25), NF-H, and microtubule-associated protein 2 (MAP2), as positive neuronal differentiation markers and inhibitor of DNA binding 2 (ID2) as a negative neuronal differentiation marker (26). In the early phase of SH-SY5Y neuronal differentiation, ID2 protein expression was attenuated by lactate treatment at day 3 (Fig. 3*A*). Lactate treatment did not affect NSE expression at day 3. In the late phase of SH-SY5Y neuronal differentiation around day 17, SH-SY5Y cells show phenotypic features of mature neuronal cells characterized by body clustering and connections with the neural process of near-differentiated cells (Fig. S2). Lactate treatment accelerated this neuronal feature at day 11, suggesting the promotive effects of lactate on the whole phase of neuronal differentiation. Lactate treatment exactly promoted the expression of the late-stage differentiation markers, NSE and NF-H, at differentiation day 6 and day 17, respectively (Fig. 3*B*). Moreover, we analyzed the effects of lactate on the differentiation of Neuro2A, a mouse neuroblastoma cell line, and found that lactate treatment induced increased expression of NSE at day 2 and MAP2 at days 2 and 6 (Fig. 3*C*). These results showed the promotive effects of lactate on neuronal differentiation in both human and mouse neuroblasts.

### Transcriptome analysis of lactate-treated SH-SY5Y cells and Neuro2A cells

**Figure 4:**
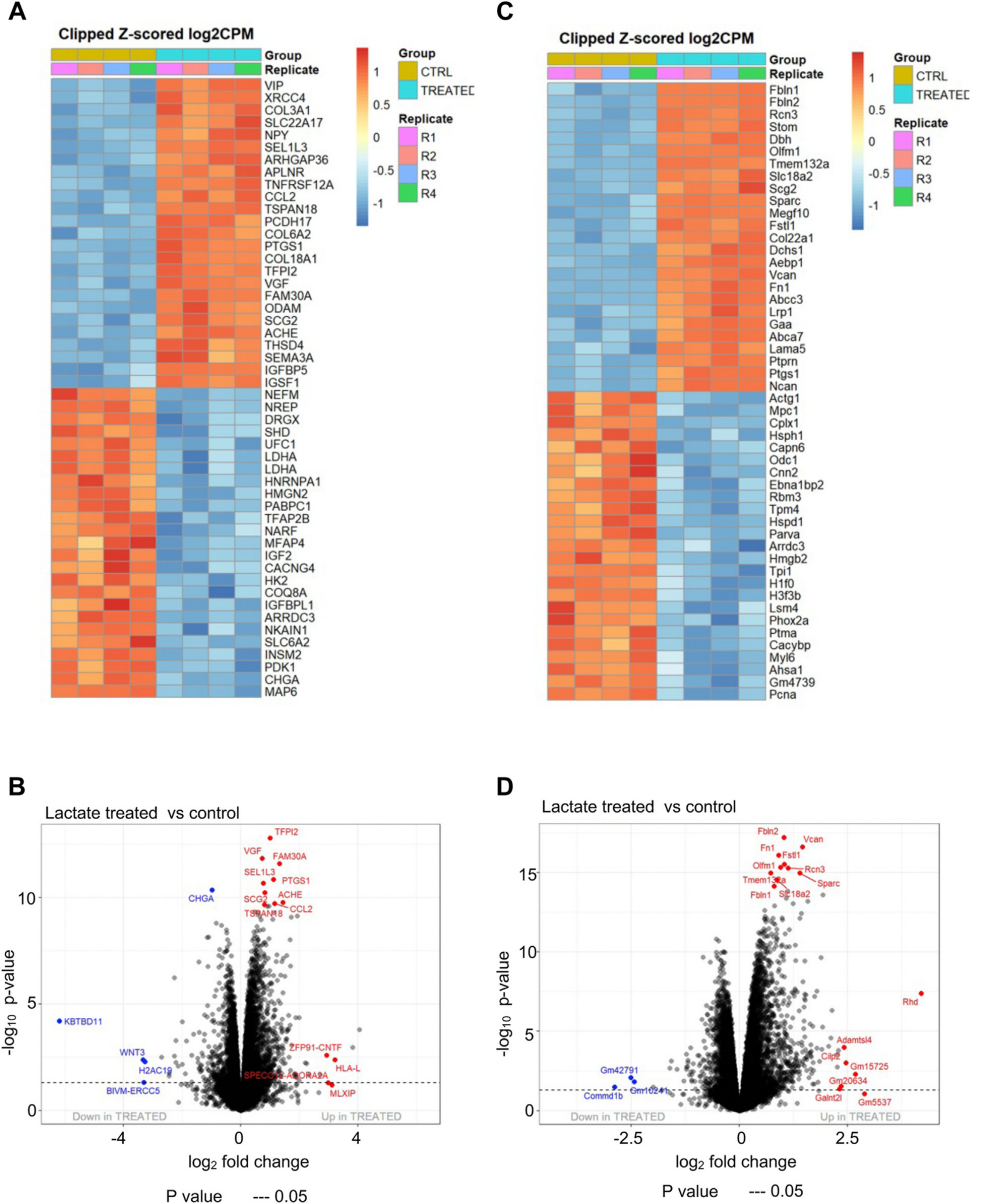
**Transcriptome analysis of lactate-treated or untreated SH-SY5Y and Neuro2A.***A*–*D*, heatmap (*A* and *C*) and volcano plots (*B* and *D*) of differentially expressed genes in lactate-treated and -untreated SH-SY5Y (*A*) and Neuro2A (*B*).

To explore the effects of lactate on comprehensive gene expressions in undifferentiated neuronal cells, we performed RNA-seq of SH-SY5Y cells and Neuro2A cells with or without 30 mM lactate treatment for 48 h. Principle component analysis (PCA) based on the entire RNA-seq showed that transcriptome profiles of the lactate-treated and -untreated cells were distinct from each other (Figs. S3*A* and S4*A*). We unidentified 1584 differentially expressed genes (DEGs) between lactate-treated and untreated SH-SY5Y cells while 6447 DEGs between lactate-treated and untreated Neuro2A cells. Heatmaps and volcano plots showed that neuronal differentiation-related genes (*CCL2* (27, 28), *SCG2* (29), *VGF* (30), *SEMA3A* (31), *NPY* (32, 33, 34, 35)) were ranked in the top 25 of lactate-induced upregulatory genes in SH-SY5Y cells (Fig. 4, *A* and *B*). Similarly, neuronal differentiation-related genes (*Scg2* (29), *Olfm1* (36), *Vcan* (37), *Fstl1* (38)) and synaptic function-related genes (*VIP* (39, 40), *Slc18a2* (41), *Lama5* (42), *Lrp1* (43)) were ranked in top 25 of lactate responsive genes in Neuro2A cells (Fig. 4, *C* and *D*). Pathway analysis indicated that signaling by activin which promotes neural differentiation (44, 45, 46) and laminin interactions responsible for neurite outgrowth (47) were upregulated in lactate-treated SH-SY5Y cells (Fig. S3*B*) and Neuro2A (Fig. S4*B*), respectively. On the other hand, the pathways of binding of TCF LEF CTNNB1 to target gene promoters which suppresses neural differentiation (48) and the pathway of signaling by FGFR2 which inhibits neuronal migration and spine density (49) were downregulated in lactate-treated SH-SY5Y (Fig. S3*C*) and Neuro2A (Fig. S4*C*). Moreover, we performed scatter plot analysis to compare the response profile to lactate on SH-SY5Y and Neuro2A and found that these cells have a significant correlation of Z-scores (Fig. S5*A*), indicating the similarities of gene expression profiles in human and mouse neuroblastoma cell lines under lactate treatment. We also found that pathway of laminin interactions which regulates neurite outgrowth (47, 50) was commonly upregulated while signaling by robo receptors (51) and regulation of expression of slits and robos (52) which regulate axon guidance were downregulated in lactate-treated SH-SY5Y cells and Neuro2A (Fig. S5*B*).

**Figure 5:**
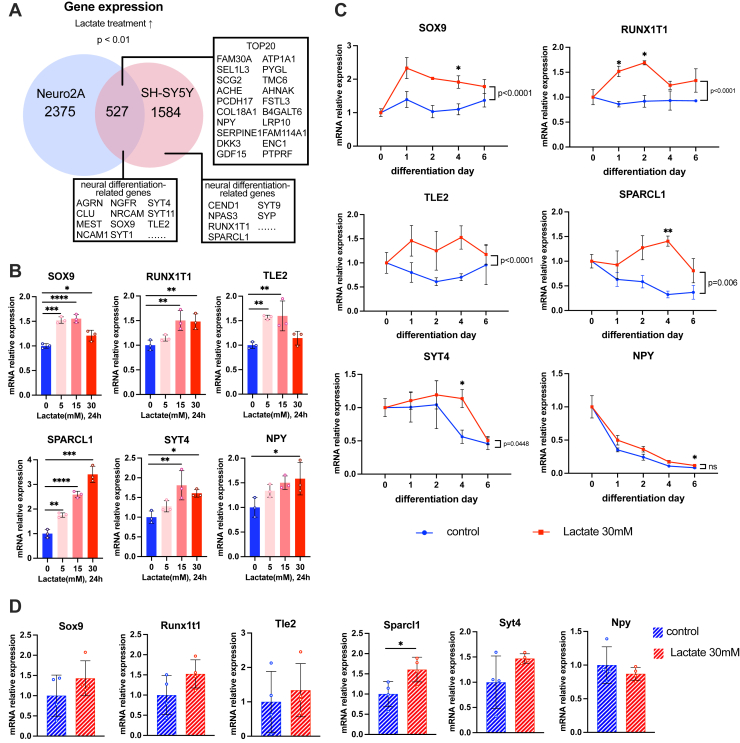
**The gene sets of lactate responsive-, neuronal differentiation-related genes in undifferentiated and differentiated SH-SY5Y cells.***A*, Venn diagram of upregulated genes upregulated by lactate treatment shared with SH-SY5Y and Neuro2A. *B*, the effects of lactate treatment on mRNA expression of neuronal differentiation-related genes in SH-SY5Y (n = 3). ∗*p* < 0.05, ∗∗*p* < 0.01, ∗∗∗*p* < 0.001, ∗∗∗∗*p* < 0.0001, *versus* control. Statistical significance was determined by one-way ANOVA followed by Dunnett tests. *C*, the effects of 30 mM lactate on mRNA expression of neuronal differentiation-related genes during differentiation of SH-SY5Y (n = 3). ∗*p* < 0.05 *versus* control at the same time point. Statistical significance was determined by one-way ANOVA followed by Dunnett tests. *p*-value *versus* control during differentiation was determined by two-way ANOVA followed by Sidak's multiple comparison tests. *D*, the effects of 30 mM lactate on mRNA expression of neuronal differentiation-related genes in primary embryonic mouse neuronal cells (n = 4). ∗*p* < 0.05, *versus* control. Statistical significance was determined by an unpaired two-tailed *t* test. Following densitometric quantification, each gene expression values were normalized to corresponding values of *RPL13A* for human or *Rpl13a* for mouse and presented as fold change to control. All data are reported as means ± SEM.

We next identified that 527 genes were commonly upregulated by lactate stimulation in both SH-SY5Y and Neuro2A (Fig. 5*A*). *NPY* (32, 33, 34, 35) and *SCG2* (29), important differentiation markers of neural cells, were ranked in the top 20 of upregulated genes by lactate. Neuronal differentiation-related genes including *AGRN* (53), *SOX9* (54, 55), *TLE* (56, 57), *MEST* (58), *CLU* (59), *NCAM1* (60), *NGFR* (61), *NRCAM* (62, 63), *SYT1* (64), *SYT4* (65), and *SYT11* (66) were also upregulated in both SH-SY5Y and Neuro2A under lactate treatment. Other crucial neural differentiation regulators such as *RUNX1T1* (67, 68), *SPARCL1* (69), *NPAS3* (70), *CEND1* (71), *SYT9* (72), and *SYP* (73) were upregulated only in SH-SY5Y cells. Based on previous reports about the functional contribution in the neural differentiation and the expression specificity of our RNA-seq data, we picked up six neural differentiation-related genes (SRY-box transcription factor 9 (*SOX9*), transducin-like enhancer of split 2 (*TLE2*), neuropeptide Y (*NPY*), synaptotagmin 4 (*SYT4*), RUNX1 partner transcriptional co-repressor 1 (*RUNX1T1*), and SPARC like 1 (*SPARCL1*) and analyzed this gene expression in 5, 15, or 30 mM lactate-treated SH-SY5Y cells for 24 h (Fig. 5*B*). The mRNA expression of *SOX9*, *RUNX1T1*, *SPARCL1*, and *SYT4* was dose-dependently promoted by lactate treatment. *TLE2* expression was increased under 5 and 15 mM lactate conditions, and *NPY* expression was increased under 30 mM lactate condition, respectively. We further analyzed this gene expression in the early stages of SH-SY5Y cell differentiation from day 1 to day 6 with or without 30 mM lactate. *SOX9*, *RUNX1T1*, *TLE2*, *SPARCL1*, and *SYT4* mRNA expression were significantly increased during differentiation (Fig. 5*C*). The gene expression of *RUNX1T1* on days 1 and 2, *SOX9*, *SPARCL1*, and *SYT4* on day 4, and *NPY* on day 6 was upregulated during the course of differentiation. Furthermore, we analyzed the effects of lactate on the identified lactate responsive genes in primary embryonic mouse neuronal cells (Fig. 5*D*). Lactate treatment significantly increased mRNA expression of *Sparcl1* but not *Sox9*, *Runx1t1*, *Tle2*, and *Syt4* in the primary embryonic mouse neuronal cells. Collectively, these results indicate that lactate comprehensively affects neuronal differentiation-related gene expression profiles and pathways in both SH-SY5Y and Neuro2A cells, and several lactate responsive genes were shared among human and mouse neuronal cell lines and primary neuronal cells.

### Lactate responsive-, neuronal differentiation-related genes are independent of G protein-coupled receptor 81 signaling

**Figure 6:**
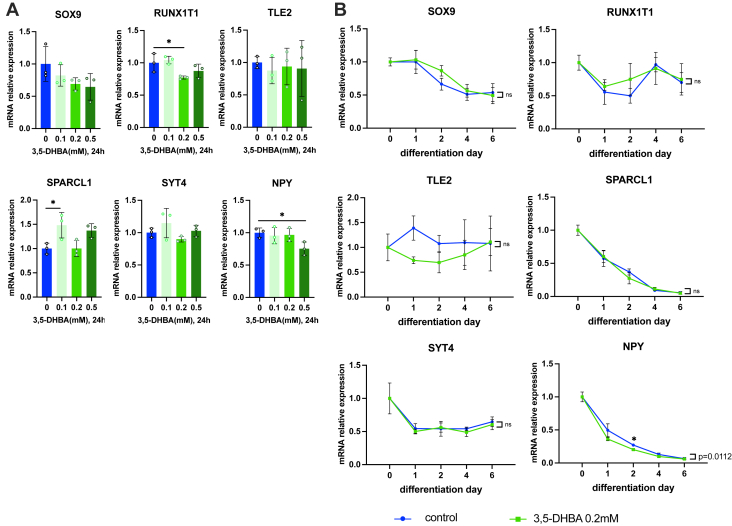
**GPR81 activation is not involved in the lactate-induced expression of neuronal differentiation genes.***A*, the effects of 3,5-DHBA, a GPR81 agonist, on neuronal differentiation-related genes in SH-SY5Y (n = 3). ∗*p* < 0.05 *versus* control. Statistical significance was determined by one-way ANOVA followed by Dunnett tests. *B*, the effects of 3,5-DHBA on mRNA expression of neuronal differentiation-related genes during differentiation of SH-SY5Y (n = 3). ∗*p* < 0.05 *versus* control at the same time point. Statistical significance was determined by one-way ANOVA with Dunnett tests. Statistical significance *versus* control during differentiation was determined by two-way ANOVA with Sidak's multiple comparison tests. Following densitometric quantification, each gene expression value was normalized to corresponding *RPL13A* values and presented as fold change to control. All data are reported as means ± SEM.

Previous studies show that lactate activates signaling cascades through lactate binding to G protein-coupled receptor 81 (GPR81) (74), cell surface receptor activating intracellular kinase signaling or lactate transport into the cytoplasm *via* MCT (17). To examine the involvement of GPR81 in the upregulation of lactate-responsive genes, we stimulated SH-SY5Y cells with 0.1, 0.2, or 0.5 mM 3,5-dihydroxybenzoic acid (3,5-DHBA), a selective agonist of GPR81, for 24 h (Fig. 6*A*). Only *SPARCL1* expression was upregulated by 3,5-DHBA treatment while *RUNX1T1* and *NPY* expression were significantly downregulated by 3,5-DHBA. We also examined the expression of lactate responsive-, neuronal differentiation-related genes in the early stage of neural differentiation of SH-SY5Y cells with or without 0.2 mM 3,5-DHBA and found that only *NPY* mRNA expression was significantly decreased during differentiation and partially downregulated at differentiation day 2 (Fig. 6*B*). Other genes were not affected by 3,5-DHBA during neuronal differentiation. Therefore, the effects of lactate on neuronal differentiation are not mediated through GPR81.

### MCT1 blocker increases the intercellular levels of lactate concentration in SH-SY5Y

**Figure 7:**
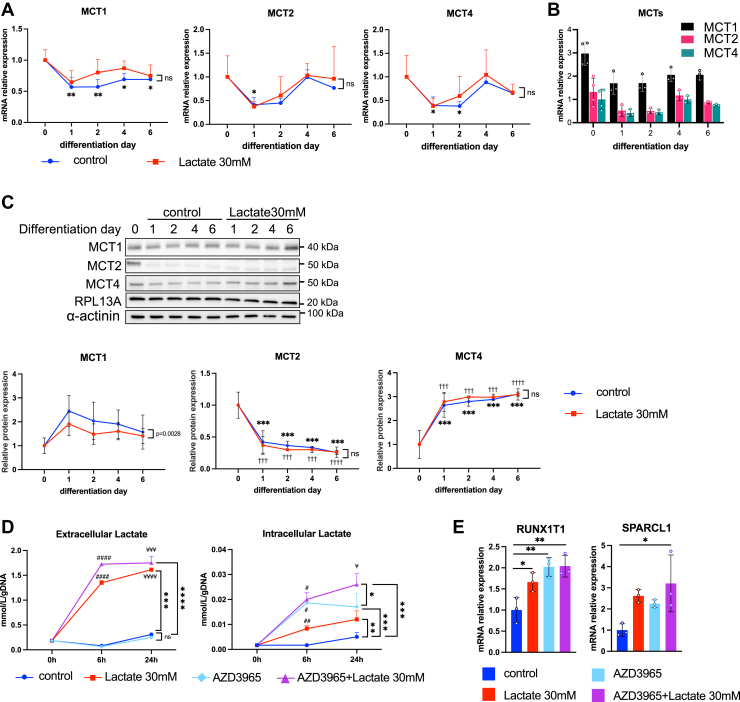
**MCT1 blocker increases intercellular lactate levels and lactate-responsive gene expression in SH-SY5Y.***A* and *C*, the effects of lactate on mRNA (*A*) and protein (*C*) expression of MCT isoforms during differentiation of SH-SY5Y (n = 3). Statistical significance *versus* control during the differentiation was determined by ANOVA followed by Sidak's multiple comparison tests. ∗*p* < 0.05, ∗∗*p* < 0.01, ∗∗∗*p* < 0.001 ∗∗∗∗*p* < 0.0001 *versus* control-day 0, ^†††^*p* < 0.001, ^††††^*p* < 0.0001, *versus* lactate 30 mM-day 0. Statistical significance was determined by one-way ANOVA followed by Dunnett tests. *B*, absolute expression levels of MCTs during neuronal differentiation of SH-SY5Y. *D*, extracellular and intracellular lactate levels of lactate- and AZD3965-, a specific MCT1 blocker, treated SH-SY5Y (n = 3). ∗*p* < 0.05, ∗∗*p* < 0.01, ∗∗∗*p* < 0.001, ∗∗∗∗*p* < 0.0001 *versus* control during treatment. Statistical significance was determined by two-way ANOVA followed with Sidak's multiple comparison tests. ^#^*p* < 0.05, ^##^*p* < 0.01, ^####^*p* < 0.0001 *versus* control-6 h, ^¥^*p* < 0.05, ^¥¥¥^*p* < 0.001, ^¥¥¥¥^*p* < 0.0001 *versus* control-24 h. Statistical significance was determined by two-way ANOVA with Sidak's multiple comparison tests. *E*, the effects of AZD3965 on mRNA expression of *RUNX1T1* and *SPARCL1* of SH-SY5Y. (n = 3) ∗*p* < 0.05, ∗∗*p* < 0.01, *versus* control. Statistical significance was determined by one-way ANOVA followed by Dunnett tests. All data are reported as means ± SEM. Following densitometric quantification, each gene expression value was normalized to corresponding RPL13A values and presented as fold change to control. Relative protein levels were normalized to the levels of RPL13A and α-actinin in the same samples and presented as fold change to control.

To examine the involvement of MCT isoforms in lactate transportation of SH-SY5Y cells, we analyzed mRNA expression of *MCT1*, *MCT2*, and *MCT4* in the early stage of SH-SY5Y differentiation with or without 30 mM lactate treatment (Fig. 7*A*). *MCT1* gene expression was significantly decreased at differentiation day 1, and the levels were kept from day 2 to 6. *MCT2* gene expression was significantly decreased at differentiation day 1 while *MCT4* gene expression was significantly decreased at differentiation days 1 and 2. *MCT1*, *MCT2*, and *MCT4* gene expressions were not significantly changed by lactate treatment during neuronal differentiation. We analyzed the absolute levels of MCT mRNA expression and found that *MCT1* is dominantly expressed in the differentiation of SH-SY5Y cells rather than *MCT2* and *MCT4* (Fig. 7*B*). However, we found a discrepancy in the expression pattern between mRNA and protein in SH-SY5Y differentiation (Fig. 7*C*). MCT1 protein expression was downregulated by lactate treatment during neuronal differentiation. MCT2 protein expression was quickly declined on differentiation day 1 and remained at low levels from day 2 to 6, while MCT4 protein expression was increased from the onset of differentiation and was maintained from day 2 to 6. These results suggested that MCT1 was stably expressed and regulated the intracellular and extracellular transports of lactate during the differentiation of SH-SY5Y.

We then analyzed the intracellular and extracellular levels of lactate in lactate-treated SH-SY5Y cells with or without AZD3965, a selective inhibitor of MCT1, for 6 and 24 h (Fig. 7*D*). AZD3965 treatment did not affect extracellular concentrations of lactate. On the other hand, the intracellular concentration of lactate was significantly increased by AZD3965 treatment in both lactate-treated and -untreated SH-SY5Y cells. Since the expression of lactate-responsive genes was dose-dependently promoted by lactate (Fig. 5*B*), we examined the combined effects of lactate and AZD3965 on the expression of RUNX1T1 and SPARCL1. Consistently, AZD3965 significantly increased mRNA expression of *RUNX1T1* and the combined treatment with AZD3965 and lactate upregulated *RUNX1T1* and *SPARCL1* (Fig. 7*E*). These results suggested that MCT1 is mainly involved in the extracellular transport of lactate during SH-SY5Y differentiation.

### Lactate binding protein, NDRG3, is involved in the promotive effects to lactate on neuronal differentiation

**Figure 8:**
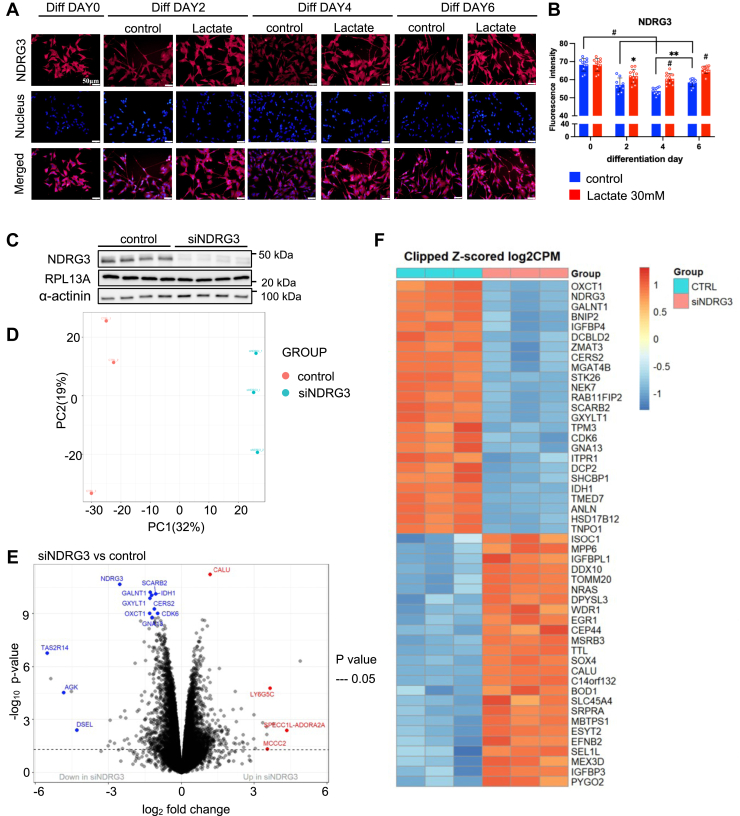
**Functional roles of NDRG3 in lactate response and neuronal differentiation in S**H**-SY5Y cells**. *A* and *B*, representative NDRG3-immunocytochemistry images of differentiated SH-SY5Y with or without lactate treatment (*A*). Scale bar = 50 μm. At least 55 cells/group were quantified from each experiment (*B*), and values were presented as mean ± SEM (n = 9–12). ∗*p* < 0.05, ∗∗*p* < 0.01, ∗∗∗*p* < 0.001, ∗∗∗∗*p* < 0.0001, *versus* control at the same time point. Statistical significance was determined by two-way ANOVA with Sidak's multiple comparison tests. ^####^*p* < 0.0001 *versus* control-day 0. Statistical significance was determined by one-way ANOVA with Dunnett tests. *C*, NDRG3 protein levels in siNDRG3- or scrambled siRNA-transfected SH-SY5Y cells. *D*–*F*, principal component analysis (PCA) (*D*), volcano plots (*E*), heatmap of differentially expressed genes (*F*) in RNA-seq of siNDRG3 or scrambled siRNA transfected SH-SY5Y cells.

**Figure 9:**
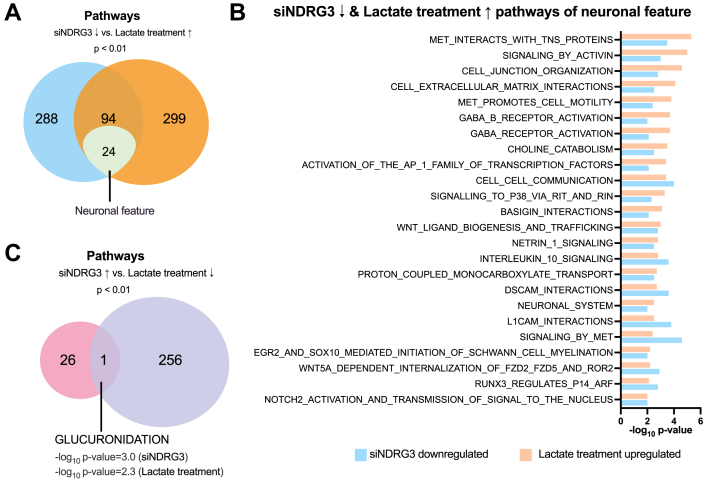
**Gene ontology (GO) term enrichment analysis of NDRG3 siRNA-treated and lactate-treated SH-SY5Y cells.** *A* and *B*, Venn diagram (*A*) and top 24 of neuronal featured pathways (*B*) which downregulated by siNDRG3 transfection and upregulated by lactate treatment. *C*, Venn diagram of upregulated pathways by siNDRG3 transfection and downregulated pathways by lactate treatment.

Previous studies showed that NDRG3 is stabilized by lactate binding and activates lactate-induced intracellular signaling pathways (75). Since lactate treatment significantly increased the intracellular levels of lactate (Fig. 1), and GPR81 agonist did not affect the mRNA expression of neuronal differentiation-related genes (Fig. 6), we analyzed the protein levels of NDRG3 during neuronal differentiation of SH-SY5Y cells with or without lactate by immunofluorescence staining (Fig. 8*A*). NDRG3 protein levels were significantly decreased during neuronal differentiation; however, lactate treatment markedly increased NDRG3 protein accumulation (Fig. 8*B*). We next performed RNA-seq of NDRG3 siRNA-transfected SH-SY5Y cells. The protein level of NDRG3 significantly decreased by NDRG3-specific siRNA transfection for 48 h (Fig. 8*C*). PCA plot showed that the transcriptome profile of siNDRG3-and control siRNA-transfected cells was distinct from each other (Fig. 8*D*). Volcano plots and heatmaps showed that NDRG3 expression was highly and specifically suppressed by NDRG3 siRNA transfection (Fig. 8, *E* and *F*). Notably, neuron development-related genes (*NEK7* (76), *SCARB2* (77), *TPM3* (78, 79), *HSD17B12* (80), *TNPO1* (81)) were ranked in the top 25 downregulated genes by NDRG3 knockdown. While *IGFBP3* (82), which inhibits neuronal differentiation, was ranked in top 25 upregulated genes by NDRG3 knockdown. The top 20 upregulated signaling pathway included CREB3 factors activate genes (Fig. S6*A*), which is a representative negative regulating pathway of astrocyte differentiation (83). To further verify the signaling pathways correlated with the lactate-NDRG3 axis, we analyzed the shared pathways between downregulated pathways in NDRG3 knockdown and upregulated pathways in lactate treatment (Fig. 9*A*). Of 94 shared pathways, 24 pathways are involved in neuronal features including neuronal system, GABA and GABA B receptor activation (84, 85), cell junction organization (86), signaling by met (87), and signaling to p38 *via* RIT and RIN (88) (Fig. 9*B*). On the other hand, glucuronidation was the only pathway that overlapped with 26 upregulated pathways in NDRG3 siRNA transfection and 256 down-regulated pathways in lactate treatment (Fig. 9*C*). In parallel, met interacts with tns proteins (89) and L1CAM interactions (90, 91), which regulate regenerative axon sprouting and synaptic plasticity, DSCAM interactions (92), which regulates neurite outgrowth, signaling by met (87) regulating neuron dendritic growth, and synaptogenesis were ranked in top 20 (Fig. S6*C*). These pathway features suggest that the promotive effect of lactate on neural differentiation are partly mediated through NDRG3 stabilization by lactate.

### TEAD1 and ELF4 are the specific transcription factors that are regulated by both lactate and NDRG3 in neuronal differentiation

**Figure 10:**
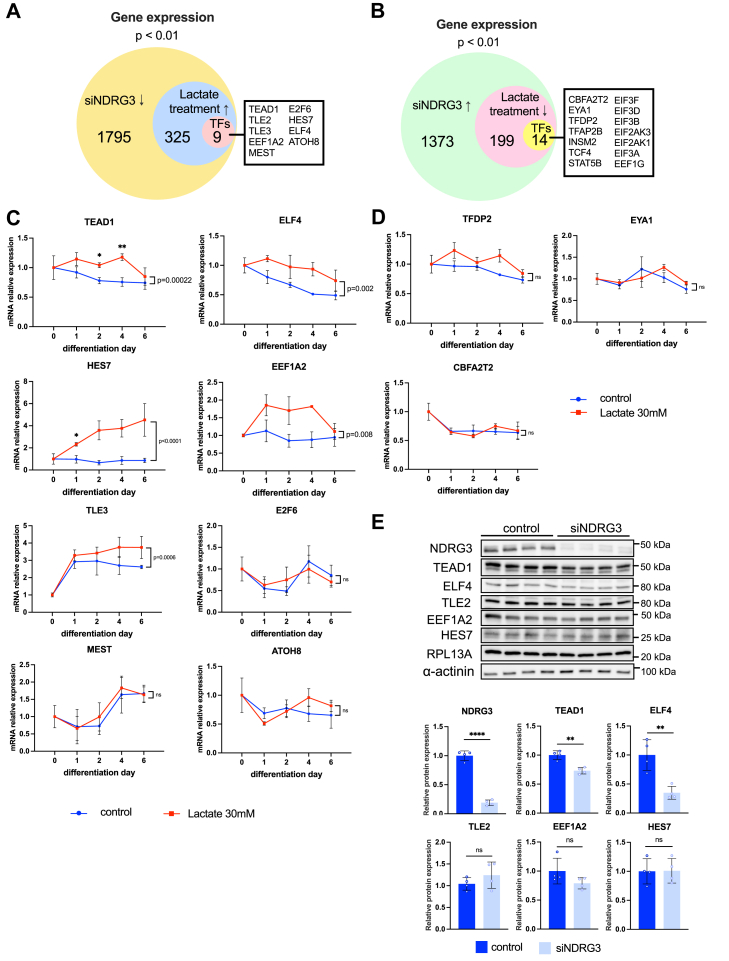
**TEAD1 and ELF4 are candidate transcription factors regulated by both NDRG3 and lactate.***A*, Venn diagram of downregulated genes by siNDRG3 transfection and upregulated genes by lactate treatment. *B*, Venn diagram of upregulated genes by siNDRG3 transfection and downregulated genes by lactate treatment. *C* and *D*, the gene expression of lactate- and NDRG3-related transcription factors during SH-SY5Y differentiation with or without 30 mM lactate treatment (n = 3, ∗*p* < 0.05, ∗∗*p* < 0.01). All data are reported as means ± SEM. Following densitometric quantification, each gene expression value was normalized to corresponding RPL13A values and presented as fold change to control (n = 3). ∗*p* < 0.05, ∗∗*p* < 0.01 *versus* control at the same time point. Statistical significance was determined by one-way ANOVA with Dunnett tests. *p*-value *versus* control during differentiation was determined by two-way ANOVA followed by Sidak's multiple comparison tests. *E*, the protein expression levels of TEAD1, ELF4, TLE2, EEF1A2, and HES7 in siNDRG3 or scrambled siRNA transfected SH-SY5Y cells (n = 4). ∗*p* < 0.05, ∗∗*p* < 0.01, ∗∗∗*p* < 0.001, ∗∗∗∗*p* < 0.0001 *versus* control. Statistical significance was determined by an unpaired two-tailed *t* test. Relative protein levels were normalized to the levels of RPL13A and α-actinin in the same samples and presented as fold change to control. Since these protein expression levels were analyzed by the same lysate in Figure 8*C*, the blots of RPL13a, α-actinin, and NDRG3 were the same and used for the normalization.

To gain further insight into downstream molecular mechanisms in the lactate–NDRG3 axis, we focused on transcription factors that were downregulated by NDRG3 knockdown and upregulated by lactate treatment. Of 1795 downregulated genes by NDRG3 knockdown, 325 genes were upregulated by lactate. Of these, we found nine transcription factors including *TEAD1*, *TLE2*, *TLE3*, eukaryotic translation elongation factor 1 alpha 2 (*EEF1A2*), E2F transcription factor 6 (*E2F6*), mesoderm specific transcript (*MEST*), Hes family BHLH transcription factor 7 (*HES7*), ETS-related transcription factor 4 (*ELF4*), and atonal BHLH transcription factor 8 (*ATOH8*) (Fig. 10*A*). Lactate treatment significantly promoted mRNA expression of *TEAD1*, *ELF4*, *TLE2*, *TLE3*, *EEF1A2*, and *HES7* (Figs. 5*B* and 10*C*) during neuronal differentiation. However, *E2F6*, *MEST*, and *ATOH8* expressions were not changed by lactate treatment. *ATOH8* mRNA expression was partially downregulated by lactate at day 1. On the other hand, of 1373 upregulated genes by NDRG3 knockdown, 199 genes were downregulated by lactate treatment. Of these, we found 14 transcription factors (*CBFA2T1*, *EYA1*, *TFDP2*, *TFAP2B*, *INSM2*, *TCF4*, *STAT5B*, *EIF3F*, *EIF3D*, *EIF3B*, *EIF2AK3*, *EIF2AK1*, *EIF3A*, *EEF1G*) (Fig. 10*B*). We analyzed three candidate genes (*CBFA2T2* (93), *EYA1* (94), *TFDP2* (95)), which were previously reported to be involved with neuron features. However, lactate treatment did not affect this gene expression during the neuronal differentiation of SH-SY5Y cells (Fig. 10*D*). Next, we examined the relevance of NDRG3 levels and the protein levels of TEAD1, ELF4, TLE2, EEF1A2, and HES7 in SH-SY5Y cells. NDRG3 knockdown significantly decreased TEAD1 and ELF4 protein levels (Fig. 10*E*). Conversely, TLE2, EEF1A2, and HES7 protein expression were not affected by NDRG siRNA transfection.

**Figure 11:**
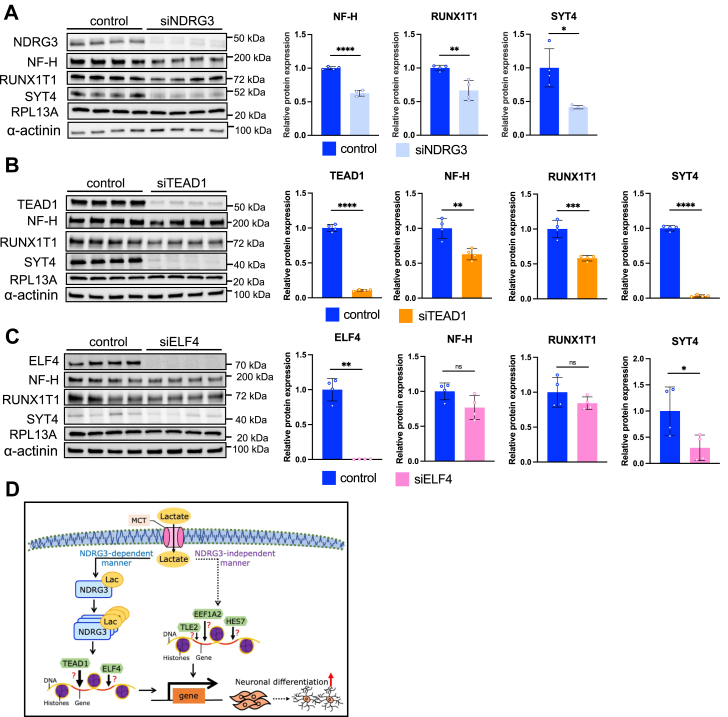
**TEAD1 and ELF4 differently affect lactate-NDRG3-regulated neuronal maker gene expressions in SH-SY5Y cells**. *A*–*C*, the protein expression levels of NF-H, RUNX1T1 and SYT4 in siNDRG3 (*A*), siTEAD1 (*B*), and siELF4 (*C*) or scrambled siRNA transfected SH-SY5Y cells (n = 4). Relative protein levels were normalized to the levels of RPL13A and α-actinin in the same samples and presented as fold change to control. All data are reported as means ± SEM. ∗*p* < 0.05, ∗∗*p* < 0.01, ∗∗∗*p* < 0.001, ∗∗∗∗*p* < 0.0001 *versus* control. Statistical significance was determined by unpaired two-tailed *t* test. Since these protein expression levels were analyzed by the same lysate in Figure 8*C*, the blots of RPL13a, α-actinin, and NDRG3 were the same and used for the normalization. *D*, a schematic illustration of lactate-induced neuronal differentiation mechanism through NDRG3-dependent and -independent signaling axis.

To further explore the functional contributions of TEAD1 and ELF4 to the lactate–NDRG3 axis in neuronal cells, we analyzed the expression of lactate-regulated neuronal maker genes in NDRG3-, TEAD1-, and ELF4-knockdown SH-SY5Y cells. The protein levels of NF-H, RUNX1T1, and SYT4 were significantly decreased by NDRG3 knockdown (Fig. 11*A*). Similarly, TEAD1 knockdown significantly decreased the protein levels of NF-H, RUNX1T1, and SYT4 (Fig. 11*B*). However, ELF4 knockdown only decreased the protein level of SYT4 (Fig. 11*C*). These results suggest that the lactate–NDRG3 signaling axis is mediated *via* TEAD1 and ELF4 in SH-SY5Y cells.

## Discussion

Lactate has been regarded as an alternative energy source in the nervous system (3); however, the extensive effects of lactate on excitability (96, 97, 98), plasticity (7), and memory consolidation (7, 99) suggest the significance of lactate-induced physiological events in the brain. Our study shows that lactate positively regulates the differentiation of human and mouse neuroblasts. We have identified novel NDRG3-dependent and -independent signaling axis, pathways, and transcription factors in neuronal cells (Fig. 11*D*). These results highlight the important roles of extracellular and intracellular lactate as critical signaling molecules that can modify neuronal functions.

One of our important findings is that lactate-induced NDRG3 stabilization promoted neuronal differentiation (Figs. 2 and 8*A*). NDRG3 is usually degraded in prolyl hydroxylase domain (PHD)/von Hippel-Lindau (VHL)-dependent manner; however, the lactate binding to NDRG3 inhibits PHD/VHL-induced ubiquitylation of NDRG3 and stabilizes NDRG3 protein (75). Despite the highest expression of NDRG3 in the cerebral cortex and the spinal cord (100), the function of NDRG3 within the nervous system has not been clarified. NDRG3 is mainly detected in neurons, mostly in excitatory, cholinergic, and serotonergic neurons (101). The expression levels of NDRG3 arise around embryonic day 9.5 (E9.5) (102) and gradually increase from E14 to postnatal day 14 in mice (103). The same expression pattern of NDRG3 can be seen in human brain samples ranging from the early prenatal period to adulthood (104). Interestingly, lactate, a regulator of NDRG3, is accumulated in fetal blood and the fetus brain during the perinatal period (13, 105). Lactate level in the E18 cortex is six-fold higher than that in the adult brain (106). Our results showed that NDRG3 protein levels were significantly decreased during *in vitro* model of neuronal differentiation, but lactate treatment markedly increased NDRG3 protein accumulation in all courses of differentiation (Fig. 8, *A* and *B*). Considering the immature blood–brain barrier (BBB) during embryonic development and different expression pattern of MCT isoforms compared with adult BBB (107), extracellular fluid-derived lactate and levels of lactate in the fetal brain may regulate neuronal differentiation through lactate-NDRG3 signaling. On the contrary, lactate level is rapidly decreased in the brain and in the circulation after delivery (108), suggesting that the lactate–NDRG3 axis is mainly involved in the early stage of neuronal differentiation during embryonic development. Based on these findings, it seems that the lactate–NDRG3 axis regulates the neuronal differentiation process at an immature embryonic stage and probably at the mature adult stage when the astrocyte lactate shuttle is established. Thus, lactate may work as a central modulator of neuronal differentiation in the embryonic state and possibly one of the mechanisms regulating neuronal plasticity. Moreover, we suggest that for *in vitro* model of neuronal differentiation using cultured cells, supplementation of lactate and the resulting stabilization of NDRG3 needs to be considered.

Previous studies reported that MCTs have a cell-specific distribution in the neural system. MCT1 is expressed in endothelial cells (109), ependymocytes (110), astrocytes (111), glia (112), and neurons (107, 109, 111, 112). MCT2 is predominantly expressed in neuronal cells and MCT4 expression appears to be specific for astrocytes (4). The cultured model of mouse cortical neurons showed low to moderate expression of MCT1 and high expression of MCT2 (111). Conversely, a high level of MCT1 expression is observed in the cultured models of hypothalamic glia and neurons (112), and developing cerebral cortex and ventrolateral hypothalamus during the late embryonic period (107). Our results showed that MCT1 was dominantly expressed in undifferentiated and differentiated SH-SY5Y cells and MCT2 expression was drastically decreased from the onset of differentiation and remained low (Fig. 7, *A*–*C*), indicating that MCT1 mainly regulates lactate transportation in neuroblasts. Previous studies reported that MCT1 is a bidirectional transporter of lactate (113, 114) and the net transport rate of MCT1 depends on the lactate gradient between efflux and influx (115). We showed that the treatment of the MCT1 blocker increased the intracellular lactate levels (Fig. 7*D*) and mRNA expression of lactate-responsive genes (Fig. 7*E*). Overall, our result indicates that the amount of cytoplasmic lactate is stable in SH-SY5Y itself but is affected by an active transport depending on the extracellular levels of lactate. These data also suggest the involvement of astrocyte-neuron lactate shuttle and microfluid-derived lactate in neuronal differentiation.

In contrast to our study, a previous study showed that suppression of lactate production by radial glial progenitors (RGPs) induces the differentiation of RGPs into neurons by impaired maintenance of mitochondrial length (10). It is worth mentioning that neural stem cells (NSCs) have multiple steps including the proliferation of NSCs, the differentiation into neural progenitor cells (NPCs), and maturation into functional neurons. RGPs are derived from neural progenitor cells and have multipotency to differentiate into both neuroblasts and glioblasts (116, 117). We used SH-SY5Y cells and Neuro2A cells, which are defined as neuroblasts, as *in vitro* models. We postulate that the effects of lactate on neuronal differentiation differ among the stage of neurogenesis. It is reasonable that neural stem cells and neuronal progenitor cells are necessary to achieve the balance of proliferation and differentiation. Lactate may regulate the course of neuronal differentiation by both positive and negative feedback loops. We also hypothesize that the roles of lactate in neuronal differentiation depend on metabolic state. Since our data showed that the amount of cytoplasmic lactate is stable during the differentiation of SH-SY5Y (Fig. 1*B*), the metabolic state of SH-SY5Y during differentiation seems mainly to consume lactate rather than to produce lactate. Therefore, autocrine/paracrine lactate regulation may not be involved in SH-SY5Y cells. Integrated neuronal circuits require cellular metabolic remodeling from glycolysis to aerobic metabolism. Mitochondrial oxidative phosphorylation is critically involved in the maturation of neural stem cells into neurons. Given that lactate is the preferred mitochondrial energy substrate in neurons after conversion into pyruvate, it seems that lactate positively or negatively regulates neural differentiation depending on the differentiation phase and energy demands.

We revealed that 24 neuronal featured pathways were co-regulated by NDRG3 and lactate stimulation (Fig. 9*B*). These pathways extensively regulate neuronal differentiation (44, 46, 88, 118, 119, 120, 121, 122, 123), neuron migration (124, 125), neurite outgrowth (92, 126), regeneration of axon (87, 89), and synaptic plasticity (90, 91, 126, 127). Pathway analysis of lactate-treated or NDRG3-knockdown SH-SY5Y cells showed the possibility of an independent regulatory mechanism through NDRG3 or lactate signaling in neuronal differentiation (Figs. 4 and 8). We further identified that TEAD1 and ELF4 are the major transcription factors in lactate-NDRG3 signal conversion (Figs. 10 and 11). TEAD1 requires cofactors to induce the transcription of target genes (128). Yes-associated protein (YAP) is one of the main coactivators of TEAD1 and regulate cell growth and organ development through the activation of Hippo pathway (129). Previous study reported that TEAD1 increases expansion of the neural progenitor population and inhibit differentiation by YAP interaction in chick embryonic fibroblasts (130). Conversely, the other study reported that TEAD1 regulates the types of neuronal differentiation through YAP-independent mechanism in the developing cortex (131). Our data showed that TEAD1 knockdown significantly inhibited SYT4, RUNX1T1, and NF-H expression in SH-SY5Y cells (Fig. 11), suggesting the direct regulation of neuronal makers by TEAD1 transcription activity. ELF4 is a member of the ETS family of transcription factors. Brain tissues have low levels of ETF4 expression (132) and the involvement of ELF4 in neuronal differentiation is currently unknown. A recent study reported that ELF4 has a functional binding site to activate the transcription of F-box protein 7 (FBXO7) (133), which has a neuroprotective role in neuronal cell death (134, 135), suggesting the possible interaction of ELF4-FBXO7 axis mediated by lactate-NDRG3 axis.

We also provided experimental evidence that lactate promoted neuronal differentiation through NDRG3-independent factors such as EEF1A2, which protects the degeneration of motor neurons (136) and dopaminergic neurons (137). Further studies are needed to elucidate the roles of these transcription factors in neuronal differentiation. Nevertheless, the extensive contribution of lactate on neuronal pathways and neuronal differentiation regulators highlights the new insight into lactate function in neuronal cells.

In conclusion, our study provides evidence that lactate functions as a cellular signaling molecule in cultured neuronal cells. Lactate promoted neuronal differentiation from the early stage *via* neuronal featured-gene expression and pathway regulations. Exogenous lactate mediated the accumulation of cellular NDRG3 protein which partially activated lactate-induced promotive phenomena in neuronal differentiation. Moreover, TEAD1 and ELF4 appear to be co-regulatory transcription factors in neuronal differentiation mediated by lactate and NDRG3. Considering the experimental results demonstrating the antidepressant effects of lactate on neuronal behavior in animal models (8, 138) and the data showing altered lactate levels in the brain of rodents (139) and humans (140) suffering neurodevelopmental disorders such as autism, schizophrenia, and bipolar disorder, our findings may contribute to elucidating lactate-regulated pathophysiological mechanisms in neuronal diseases.

## Experimental procedures

### Cell lines

SH-SY5Y, human neuroblastoma cell lines, was obtained from ATCC and initially cultured with 1 × 10^4^ cells/cm^2^ cell density in 1:1 mixture of E-MEM (051-07615, Fujifilm Wako) and Ham's F-12 (087-08335, Fujifilm Wako) containing 10% fetal bovine serum (FBS, 10437028, Gibco), and 100 mg/ml penicillin/streptomycin (168-23191, Fujifilm Wako). Neuro2A, a mouse neuroblastoma cell line, was obtained from ATCC and initially culture with 5 × 10^3^ cells/cm^2^ cell density in E-MEM with similar conditions.

### Neural differentiation of SH-SY5Y and Neuro2A

The differentiation process consisted of 11 steps spread out over the course of a 17-day period for SH-SY5Y cells. First, 1 × 10^5^ cells were seeded onto 35 mm dishes. On first day of the differentiation (day 0), the cells were cultured in Differentiation Media #1 (E-MEM supplemented with 2.5% FBS, 1% penicillin/streptomycin, 10 μM all-*trans* retinoic acid (RA) (186-01114, Fujifilm Wako). On day 6, the cells were split 1:1 onto 35 mm dishes and cultured in Differentiation Media #1. On day 7, the cell culture medium was changed to Differentiation Media #2 (E-MEM supplemented with 1% FBS, 1% penicillin/streptomycin, 10 μM RA). On day 9, the cells were split 1:1 onto MaxGelECM (E0282, Sigma-Aldrich)-coated 35 mm dishes and cultured in Differentiation Media #2. On day 10, the cells were cultured in Differentiation Media #3 (1xB-27 (17504001, ThermoFisher Scientific), 20 mM KCl, 100 mg/ml penicillin/streptomycin, 1 mM Glutamaxl (35050061, ThermoFisher Scientific), 50 ng/ml BDNF (218441-99-7, Fujifilm Wako), 2 mM dibutyryl cyclic AMP (D0260, Sigma), 10 μM RA in Neurobasal (2110304, ThermoFisher Scientific)), and maintained until day 17. To analyze the effect of lactate (L7022, Sigma-Aldrich) or 3,5-DHBA (D110000, Sigma) on the differentiation of SH-SY5Y cells, cells were treated with 30 mM lactate or 0.2 mM 3,5-DHBA respectively. To induce the differentiation of Neuro2A cells, 1 × 10^5^ cells were seeded in 60-mm dishes and incubated in E-MEM supplemented with 2% FBS, 1% penicillin/streptomycin, 20 μM RA. Cell culture medium was changed every day.

### Lactate measurement

6.0 × 10^5^ SH-SY5Y cells were cultured in 100 mm dishes and treated with 5, 15, or 30 mM lactate in the differentiation medium, or treated with 10 μM AZD3965 (1448671-31-5, Cayman Chemical) in the growth medium, respectively. Extracellular and intracellular lactate levels were measured by Lactate Assay Kit-WST (L256, DOJINDO) according to the manufacturer’s protocol. Meantime, genome DNA was isolated using Wizard SV Genomic DNA Purification System (A2360, Promega), and the concentration of lactate was normalized to the corresponding genome DNA values.

### Immunocytochemistry

SH-SY5Y cells were fixed in 4% paraformaldehyde for 20 min, and permeabilized with PBS containing 0.05% Triton X-100 and 5% goat serum for 60 min at room temperature. Permeabilization samples were incubated with primary antibodies of neurofilament-H (NF-H) (ab4680, abcam), beta III Tubulin (TUBB3) (ab78078, abcam), NDRG3 (ab133715, abcam) at 4 °C overnight. Immunostaining and nuclei were visualized by Alexa Fluor-488 (A-11001, ThermoFisher Scientific) or Alexa Fluor-555 (A-21428, ThermoFisher Scientific) fluorescence-conjugated secondary antibodies and Hoechst-33342 (H3570, ThermoFisher Scientific).

### Neurites outgrowth assay

SH-SY5Y cells were seeded in 24-well plates at a density of 2 × 10^4^ cells/well. Cells were treated with or without 30 mM lactate and cultured in a differentiation medium (n = 4). Neurites were immuno-stained with anti-NF-H (ab4680, abcam) or anti-TUBB3 (ab78078, abcam) antibodies at differentiation day 0, 4, 6 (n = 4). At least 50 cells from seven randomly selected fields were counted. Three images around the center of each well were taken using a 20× objective, and more than 1000 cells in each group were counted for neurite outgrowth. The lengths of neurites were analyzed using Image-J software with the Neuron-J plugin (National Institutes of Health). To determine the differentiation rate, a differentiated cell was defined as a cell with a neurite length greater than the 2-fold cell body of the individual cell. The neurite lengths of 1000 cells were displayed in a density plot or a histogram. Data were analyzed using Python software (3.7.10 version) and utilized the default parameter function. The median of descriptive statistics for the total neurite length of the plot, which represented both NF-H- and TUBB3-positive cells, was quantified.

### Western blotting

Cells were lysed in RIPA lysis buffer (150 mM NaCl, 1.0% Nonidet P-40, 0.5% deoxycholic acid, 0.1% SDS, 50 mM Tris (pH 8.0), 0.1% Na_3_VO_4_, and protease inhibitor mixture), and protein concentrations were determined by a BCA Protein Assay Kit (23227, ThermoFisher Scientific). Protein lysates were separated by 4 to 20% SDS-PAGE gel and transferred to nitrocellulose membranes. The membranes were blocked in 5% fat-free milk in TBST (20 mM Tris-HCl (pH 7.6), 0.15 M sodium chloride, and 0.1% Tween 20) for 1 h, washed three times with TBST, and incubated with primary antibodies that probe for NSE (9536, CST), ID2 (3431, CST), MAP2 (4542, CST), NF-H (ab4680, abcam), RPL13A (2765, CST), α-actinin (6487, CST), Cyclophilin B (43603, CST), β-actin (8457, CST), NDRG3 (ab133715, abcam), MCT1 (20139-1-AP, Proteintech), MCT2 (ab224627, abcam), MCT4 (ab74109, abcam), TEAD1 (12292, CST), ELF4 (sc-390689, Santacruz), EEF1A2 (GTX102326, GeneTex), HES7 (AP9712a, Abcepta), TLE2 (GTX106107, GeneTex), RUNX1T1 (15494-1-AP, Proteintech) and SYT4 (12642-1-AP, Proteintech) in TBST for overnight. After three washes in TBST, the membranes were incubated with horseradish peroxidase-conjugated anti-rabbit (7074, CST) or anti-mouse (7076, CST) secondary antibodies diluted 1:5000 in 5% fat-free milk in TBST for 1 h and washed three times in TBST. The blots were developed with the enhanced chemiluminescence substrate (RPN2232, GE Healthcare) according to the manufacturer's instructions and imaged with a ChemiDoc Touch System (1708370, Bio-Rad).

### RNA sequencing

The samples were quantified using an Agilent 4200 Tapestation instrument, with a corresponding Agilent High Sensitivity RNA assay. The resulting RIN (RNA Integrity Number) scores and concentrations were taken into account for qualifying samples to proceed. Poly (A) RNA preparation was performed using Poly (A) mRNA Magnetic Isolation Module (E7490, NEB). Library preparation was performed using NEBNext UltraII Directional RNA Library Prep Kit for Illumina (E7760, NEB). The pool was denatured and loaded onto a NovaSeq 6000 (Illumina), with an Illumina NextSeq High Output 150-cycle kit to obtain Paired-End 75 bp reads. The pool was loaded at 1.9 pM, with 5% PhiX spiked in to serve as a sequencing control. The resulting FASTQ files were used in subsequent analysis.

### Bioinformatics analysis

RNA-seq analysis was performed as described previously (141). To combine the Z-scores of lactate-treated *versus* control in human and mouse data, we used the gene symbols that were present in both datasets. We kept the ones that had large absolute Z-scores. To make the Z-scores comparable between datasets, we performed a Rank product test (142) to determine if both a human gene and its homologous mouse gene were highly ranked (either positively or negatively in the same direction).

### Quantitative reverse transcription PCR

Total RNA was isolated using FastGene RNA Basic Kit (FG-80050, NIPPON Genetics), and reverse transcribed with the iScript Reverse Transcription Supermix for RT-qPCR (1708841, BIO-RAD). Complementary DNA was amplified with SYBR Green Master Mix (3485612, ThermoFisher Scientific) using an StepOnePlus PCR System. Relative levels of mRNA expression were calculated by *RPL13A* for human or *Rpl13a* for mouse. Primer sequences are shown in Table S1.

### Isolation and culture of primary embryonic mouse neuronal cells

Nine-week-old C57BL/6J pregnant mice at 16.5 days postcoitum (d.p.c.) were anesthetized, and embryos at E16.5 were collected by performing a cesarean section from the dams. The fetal brain was gently pulled away from the embryo and cleaned with forceps to remove the cerebellum and associated meninges. The brain tissues were temporarily stored in Hanks' balanced salt solution without calcium or magnesium (HBSS, w/o) (084-08345, Fujifilm Wako). After collecting embryonic brains from dams, brain tissues were dissociated into single cells by using the MACS Neural Tissue Dissociation Kit (P) (130-092-628, Miltenyi Biotec), gentleMACS C tubes (130-093-237, Miltenyi Biotec), and the gentleMACS Octo Dissociator with Heaters (130-096-427, Miltenyi Biotec) according to the manufacturer's protocol and the recommended gentleMACS program (37C_NTDK_1). After the enzymatic digestion by the gentleMACS system, HBSS with calcium and magnesium (HBSS, w) (084-08965, Fujifilm Wako) was added to the cell suspension. The mixture of the cell suspension and HBSS was centrifuged at room temperature and at 300*g* for 10 min and filtered through a 70 μm cell strainer (130-098-462, Miltenyi Biotec). Typically, we dissected 7 to 10 embryos, collected 200 to 300 mg of brains, digested the tissues with approximately 2 ml of the enzymatic solution of MACS Neural Tissue Dissociation Kit (P), and mixed with 10 ml of HBSS. The filtered cell suspension was centrifuged at 300*g* for 10 min, and the cell pellet was incubated in 10 ml of cold Red Blood Cell Lysis Buffer (60-00050-12, pluriSelect) for 10 min in the refrigerator (2−8 °C), followed by centrifugation at room temperature and at 300*g* for 10 min. Finally, 99% of live cells were isolated after brain dissociation. Next, neuronal cells were purified from the dissociated single cells by magnetic-activated cell sorting using the Neuron Isolation Kit, mouse (130-115-389, Miltenyi Biotec), LS Column (130-042-401, Miltenyi Biotec), and MACS Separator (130-090-976, Miltenyi Biotec) according to the manufacturer's protocol. Approximately 53% of cells were isolated as neuronal cells from single-cell suspension by magnetic sorting. Isolated primary embryonic neuronal cells were resuspended in Neurobasal medium (21103-049, ThermoFisher Scientific) supplemented with B27 (17504-044, ThermoFisher Scientific), 2 mM Glutamax (35050-061, ThermoFisher Scientific), 25 μM β-mercaptoethanol (198-15781, Fujifilm Wako), and 100 mg/ml penicillin/streptomycin (168-23191, Fujifilm Wako) and seeded onto poly-d-lysine-coated 6-well plates (354413, Corning) at a density of 1 × 10^6^ cells per well. Three days after plating, 50% of the medium was changed. The cells were treated with 30 mM lactate for 24 h. All animal experiment were approved by Tohoku University Ethical Committee.

### RNA interference

Transfection of siRNA duplexes specific to NDRG3 (CAAACCUACAGAAUGCAUA, UAUGCAUUCUGUAGGUUUG), TEAD1 (CUCCUUUGGGAAGCAAGUA, UACUUGCUUCCCAAAGGAG), ELF4 (GCGAUAUCCUGGAUGAGAA, UUCUCAUCCAGGAUAUCGC) and control (SR30004, Origene) was performed using Lipofectamine RNAiMAX (13778-030, Invitrogen). 100,000 cells were plated onto 35 mm dishes and were transfected with 20 nM siRNAs for 48 h according to the manufacture’s instruction.

### Statistical analysis

All data are reported as means ± SEM. The normality of variables was determined by QQ-plot. Statistical significance between the two groups was determined by unpaired two-tailed *t* test, but a Welch’s *t* test was performed when *p* < 0.05 in F-test. Statistical significance between multiple groups was determined by two-way ANOVA followed by Sidak's multiple comparison tests or one-way ANOVA followed by Dunnett tests. Statistical significance was defined as *p* < 0.05. All statistical analyses were performed by Prism 9 software.

## Data availability

Further information and requests for resources of RNA-seq law data should be directed to and will be fulfilled by Joji Kusuyama (joji.kusuyama.bsin@tmd.ac.jp).

## Supporting information

This article contains supporting information.

## Conflict of interest

The authors declare that they have no conflicts of interest with the contents of this article.

## Supporting information

Supplemental Table 1 List of primer sequences used in RT-qPCR.

Supplemental Figure 1 **The proportion of the neurite length in NF-H- and TUBB3-positive SH-SY5Y cells.***A* and *B*, histogram of the neurites length of differentiated NF-H-positive (*A*) and TUBB3-positive (*B*) SH-SY5Y cells at day 4 and 6.

Supplemental Figure 2 **Morphological appearance of differentiated SH-SY5Y cells with lactate treatment.** Phase contrast images of SH-SY5Y cells at day 11, 17 of the differentiation process with or without 30 mM lactate supplementation. Scale bar = 100 μm or 200 μm.

Supplemental Figure 3 **GO term enrichment analysis of lactate-treated or untreated SH-SY5Y cells by RNA-Seq.***A*, PCA of lactate treated and untreated SH-SY5Y cells. *B*, top 20 of upregulated pathways by lactate treatment in SH-SY5Y. *C*, bottom 20 of downregulated pathways by lactate treatment in SH-SY5Y.

Supplemental Figure 4 **GO term enrichment analysis of lactate-treated or untreated Neuro2A cells by RNA-Seq.***A*, PCA of lactate treated and untreated Neuro2A cells. *B*, top 20 of upregulated pathways top 20 by lactate treatment in Neuro2A. *C*, bottom 20 of downregulated pathways bottom 20 by lactate treatment in Neuro2A.

Supplemental Figure 5 **Correlation of lactate responses between SH-SY5Y and Neuro2A.***A*, scatter plot analysis of gene expressions between lactate-treated SH-SY5Y cells and Neuro2A cells. *B*, over representation analysis (ORA) of the top 10 shared pathways of upregulated and downregulated pathways in lactate-treated SH-SY5Y cells and Neuro2A cells.

Supplemental Figure 6 **GO term enrichment analysis of siNDRG3-treated or untreated SH-SY5Y cells by RNA-Seq.***A* and *B*, top 20 of downregulated pathways (*A*) and bottom 20 of upregulated pathways (*B*) by NDRG3 knockdown in SH-SY5Y. *C*, top 20 pathways which downregulated pathways downregulated by siNDRG3 transfection and upregulated by lactate treatment.
