# Supplementary figures and images for: Lactate promotes neuronal differentiation of SH-SY5Y cells by lactate-responsive gene sets through NDRG3-dependent and -independent manners
Source: J Biol Chem. 2023 May 10;299(6):104802. doi: 10.1016/j.jbc.2023.104802 (PMC10276297; doi:10.1016/j.jbc.2023.104802)

A NF-H-positive cells

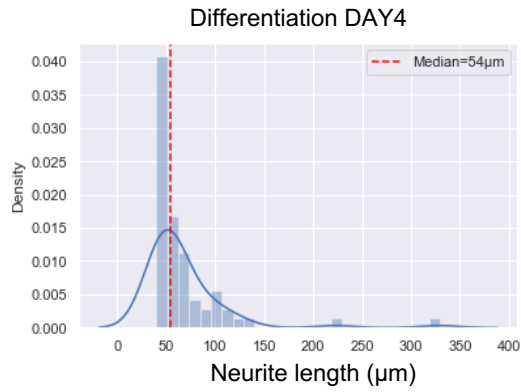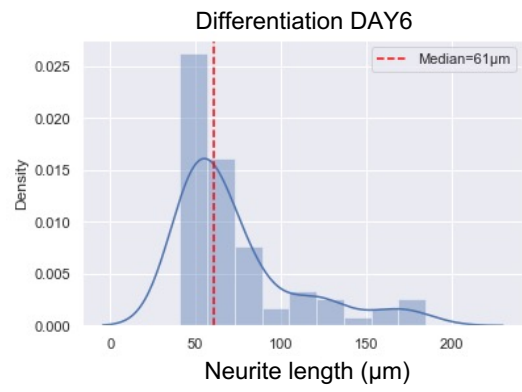

B TUBB3-positive cells

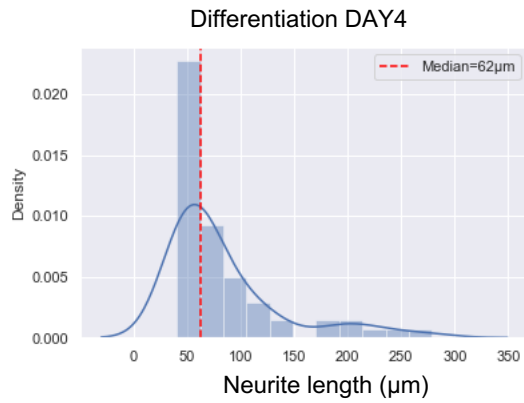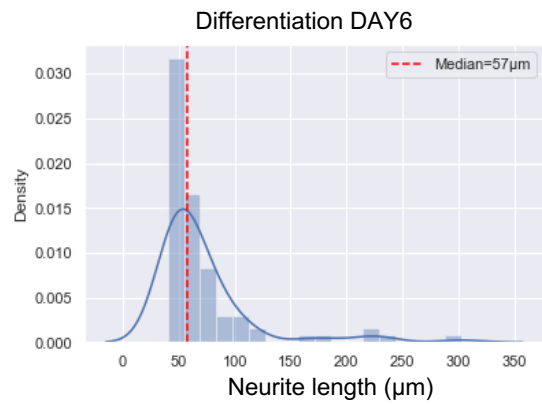

Supplement: Supplemental Figure 1 — The proportion of the neurite length in NF-H- and TUBB3-positive SH-SY5Y cells.A and B, histogram of the neurites length of differentiated NF-H-positive (A) and TUBB3-positive (B) SH-SY5Y cells at day 4 and 6. [file mmc2.pdf]

# SH-SY5Y

Differentiation DAY11

control

Lactate

X20

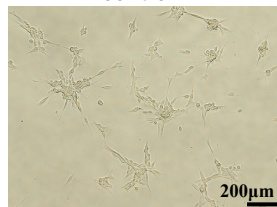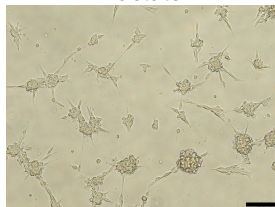

X10

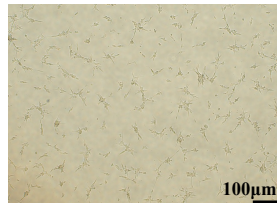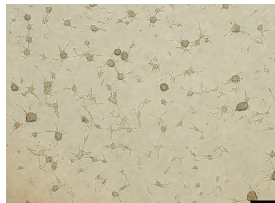

# SH-SY5Y

Differentiation DAY17

control

Lactate

X20

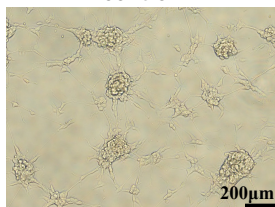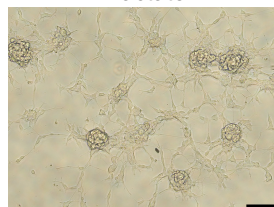

X10

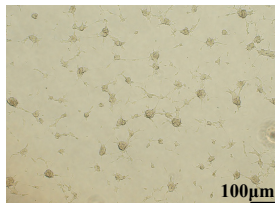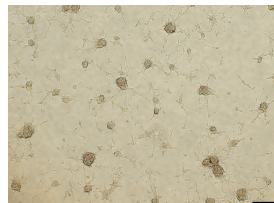

Supplement: Supplemental Figure 2 — Morphological appearance of differentiated SH-SY5Y cells with lactate treatment. Phase contrast images of SH-SY5Y cells at day 11, 17 of the differentiation process with or without 30 mM lactate supplementation. Scale bar = 100 μm or 200 μm. [file mmc3.pdf]

A

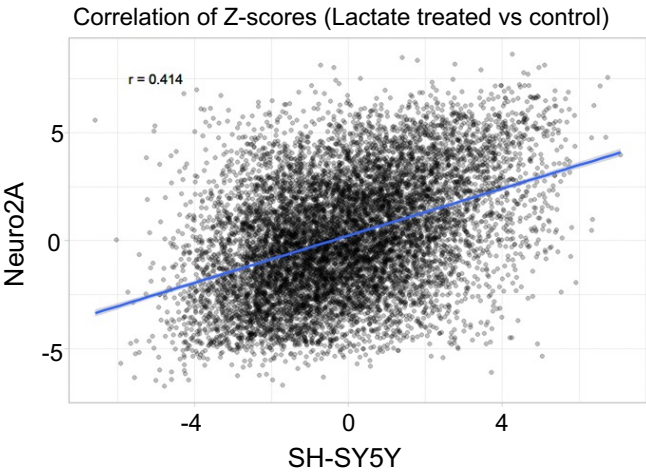

B

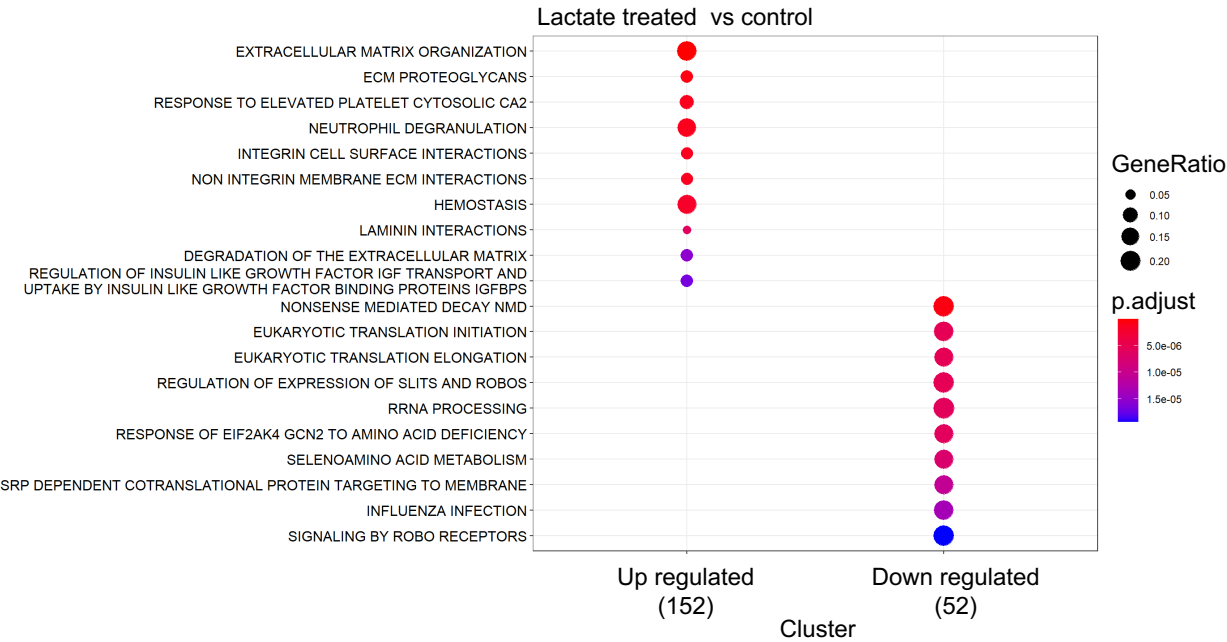

Supplement: Supplemental Figure 5 — Correlation of lactate responses between SH-SY5Y and Neuro2A.A, scatter plot analysis of gene expressions between lactate-treated SH-SY5Y cells and Neuro2A cells. B, over representation analysis (ORA) of the top 10 shared pathways of upregulated and downregulated pathways in lactate-treated SH-SY5Y cells and Neuro2A cells. [file mmc6.pdf]
