# Supplementary material for: Lactate promotes neuronal differentiation of SH-SY5Y cells by lactate-responsive gene sets through NDRG3-dependent and -independent manners
Source: J Biol Chem. 2023 May 10;299(6):104802. doi: 10.1016/j.jbc.2023.104802 (PMC10276297; doi:10.1016/j.jbc.2023.104802)
Supplement: Supplemental Figure 3 — GO term enrichment analysis of lactate-treated or untreated SH-SY5Y cells by RNA-Seq.A, PCA of lactate treated and untreated SH-SY5Y cells. B, top 20 of upregulated pathways by lactate treatment in SH-SY5Y. C, bottom 20 of downregulated pathways by lactate treatment in SH-SY5Y. [file mmc4.pdf]

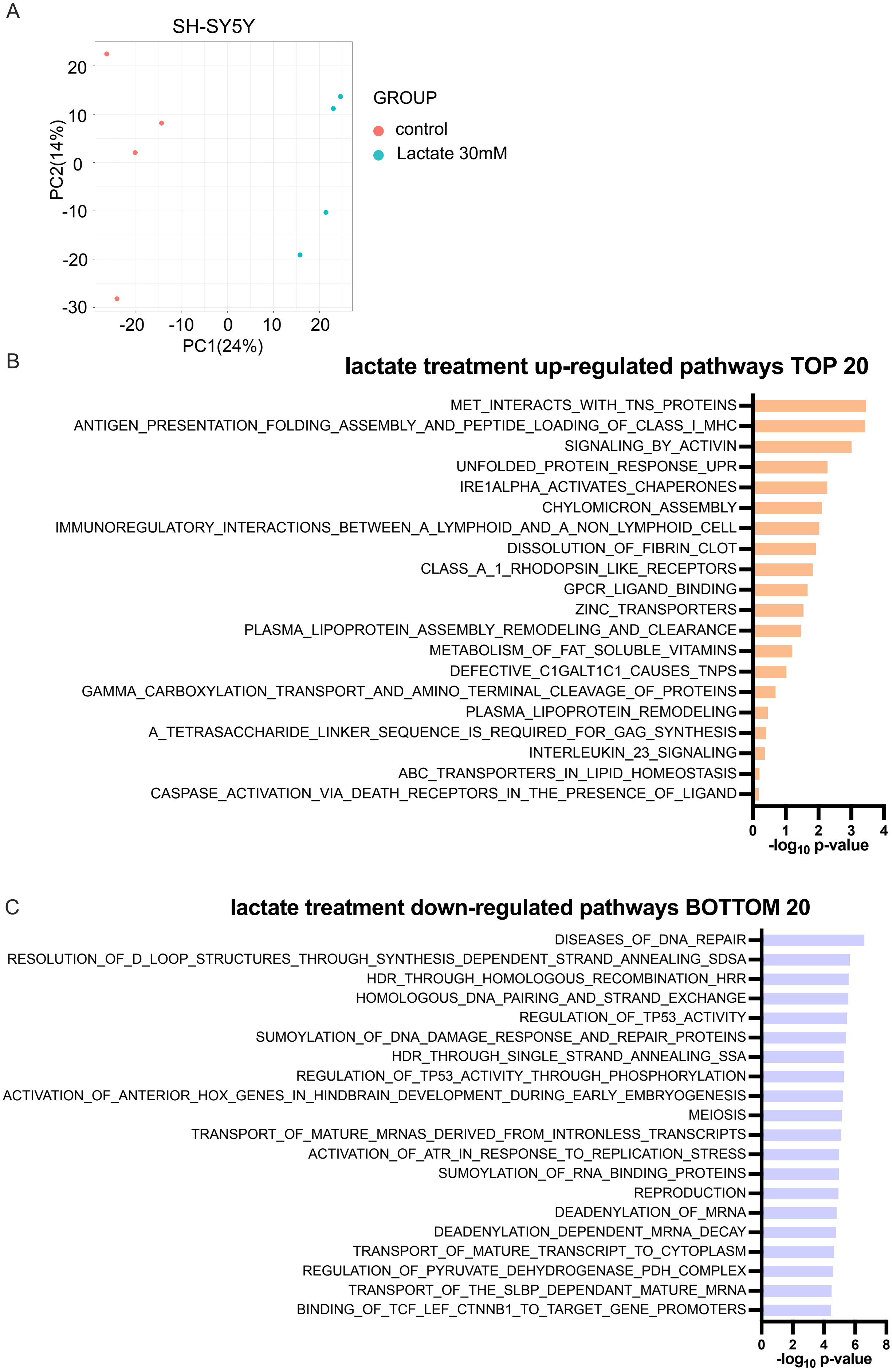
A
SH-SY5Y
PC2(14%)
20
10
0
-10
-20
-30
-20
-10
0
10
20
PC1(24%)
GROUP
control
Lactate 30mM
B
lactate treatment up-regulated pathways TOP 20
MET_INTERACTS_WITH_TNS_PROTEINS
ANTIGEN_PRESENTATION_FOLDING_ASSEMBLY_AND_PEPTIDE_LOADING_OF_CLASS_I_MHC
SIGNALING_BY_ACTIVIN
UNFOLDED_PROTEIN_RESPONSE_UPR
IRE1ALPHA_ACTIVATES_CHAPERONES
CHYLOMICRON_ASSEMBLY
IMMUNOREGULATORY_INTERACTIONS_BETWEEN_A_LYMPHOID_AND_A_NON_LYMPHOID_CELL
DISSOLUTION_OF_FIBRIN_CLOT
CLASS_A_1_RHODOPSIN_LIKE_RECEPTORS
GPCR_LIGAND_BINDING
ZINC_TRANSPORTERS
PLASMA_LIPOPROTEIN_ASSEMBLY_REMODELING_AND_CLEARANCE
METABOLISM_OF_FAT_SOLUBLE_VITAMINS
DEFECTIVE_C1GALT1C1_CAUSES_TNPS
GAMMA_CARBOXYLATION_TRANSPORT_AND_AMINO_TERMINAL_CLEAVAGE_OF_PROTEINS
PLASMA_LIPOPROTEIN_REMODELING
A_TETRASACCHARIDE_LINKER_SEQUENCE_IS_REQUIRED_FOR_GAG_SYNTHESIS
INTERLEUKIN_23_SIGNALING
ABC_TRANSPORTERS_IN_LIPID_HOMEOSTASIS
CASPASE_ACTIVATION_VIA_DEATH_RECEPTORS_IN_THE_PRESENCE_OF_LIGAND
0
1
2
3
4
$-\log_{10}$ p-value
C
lactate treatment down-regulated pathways BOTTOM 20
DISEASES_OF_DNA_REPAIR
RESOLUTION_OF_D_LOOP_STRUCTURES_THROUGH_SYNTHESIS_DEPENDENT_STRAND_ANNEALING_SDSA
HDR_THROUGH_HOMOLOGOUS_RECOMBINATION_HRR
HOMOLOGOUS_DNA_PAIRING_AND_STRAND_EXCHANGE
REGULATION_OF_TP53_ACTIVITY
SUMOYLATION_OF_DNA_DAMAGE_RESPONSE_AND_REPAIR_PROTEINS
HDR_THROUGH_SINGLE_STRAND_ANNEALING_SSA
REGULATION_OF_TP53_ACTIVITY_THROUGH_PHOSPHORYLATION
ACTIVATION_OF_ANTERIOR_HOX_GENES_IN_HINDBRAIN_DEVELOPMENT_DURING_EARLY_EMBRYOGENESIS
MEIOSIS
TRANSPORT_OF_MATURE_MRNAS_DERIVED_FROM_INTRONLESS_TRANSCRIPTS
ACTIVATION_OF_ATR_IN_RESPONSE_TO_REPLICATION_STRESS
SUMOYLATION_OF_RNA_BINDING_PROTEINS
REPRODUCTION
DEADENYLATION_OF_MRNA
DEADENYLATION_DEPENDENT_MRNA_DECAY
TRANSPORT_OF_MATURE_TRANSCRIPT_TO_CYTOPLASM
REGULATION_OF_PYRUVATE_DEHYDROGENASE_PDH_COMPLEX
TRANSPORT_OF_THE_SLBP_DEPENDANT_MATURE_MRNA
BINDING_OF_TCF_LEF_CTNNB1_TO_TARGET_GENE_PROMOTERS
0
2
4
6
8
$-\log_{10}$ p-value
