# Supplementary material for: Lactate promotes neuronal differentiation of SH-SY5Y cells by lactate-responsive gene sets through NDRG3-dependent and -independent manners
Source: J Biol Chem. 2023 May 10;299(6):104802. doi: 10.1016/j.jbc.2023.104802 (PMC10276297; doi:10.1016/j.jbc.2023.104802)
Supplement: Supplemental Figure 4 — GO term enrichment analysis of lactate-treated or untreated Neuro2A cells by RNA-Seq.A, PCA of lactate treated and untreated Neuro2A cells. B, top 20 of upregulated pathways top 20 by lactate treatment in Neuro2A. C, bottom 20 of downregulated pathways bottom 20 by lactate treatment in Neuro2A. [file mmc5.pdf]

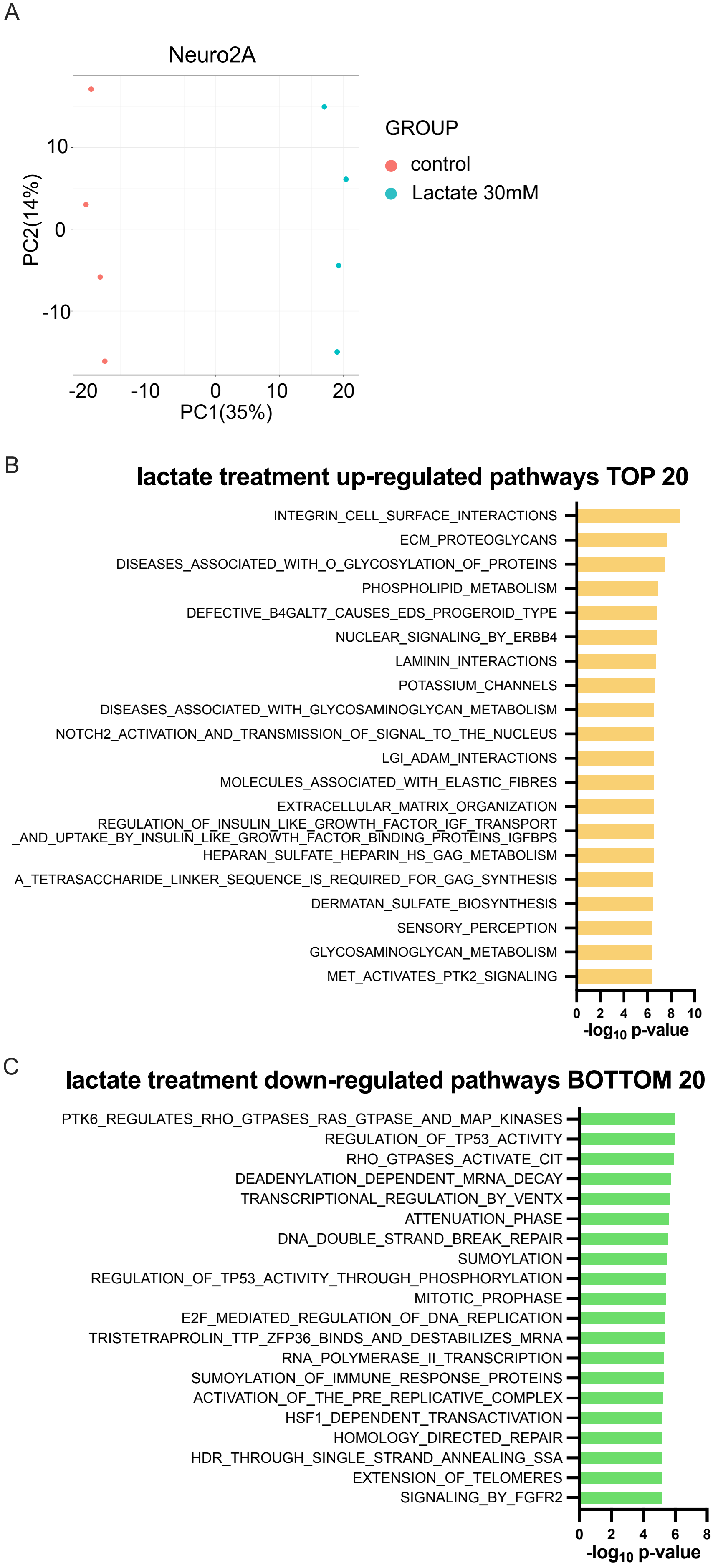
A
Neuro2A
PC2(14%)
10
0
-10
-20
-10
0
10
20
PC1(35%)
GROUP
control
Lactate 30mM
B
lactate treatment up-regulated pathways TOP 20
INTEGRIN_CELL_SURFACE_INTERACTIONS
ECM_PROTEOGLYCANS
DISEASES_ASSOCIATED_WITH_O_GLYCOSYLATION_OF_PROTEINS
PHOSPHOLIPID_METABOLISM
DEFECTIVE_B4GALT7_CAUSES_EDS_PROGEROID_TYPE
NUCLEAR_SIGNALING_BY_ERBB4
LAMININ_INTERACTIONS
POTASSIUM_CHANNELS
DISEASES_ASSOCIATED_WITH_GLYCOSAMINOGLYCAN_METABOLISM
NOTCH2_ACTIVATION_AND_TRANSMISSION_OF_SIGNAL_TO_THE_NUCLEUS
LGI_ADAM_INTERACTIONS
MOLECULES_ASSOCIATED_WITH_ELASTIC_FIBRES
EXTRACELLULAR_MATRIX_ORGANIZATION
REGULATION_OF_INSULIN_LIKE_GROWTH_FACTOR_IGF_TRANSPORT
_AND_UPTAKE_BY_INSULIN_LIKE_GROWTH_FACTOR_BINDING_PROTEINS_IGFBPS
HEPARAN_SULFATE_HEPARIN_HS_GAG_METABOLISM
A_TETRASACCHARIDE_LINKER_SEQUENCE_IS_REQUIRED_FOR_GAG_SYNTHESIS
DERMATAN_SULFATE_BIOSYNTHESIS
SENSORY_PERCEPTION
GLYCOSAMINOGLYCAN_METABOLISM
MET_ACTIVATES_PTK2_SIGNALING
0 2 4 6 8 10
$-\log_{10}$ p-value
C
lactate treatment down-regulated pathways BOTTOM 20
PTK6_REGULATES_RHO_GTPASES_RAS_GTPASE_AND_MAP_KINASES
REGULATION_OF_TP53_ACTIVITY
RHO_GTPASES_ACTIVATE_CIT
DEADENYLATION_DEPENDENT_MRNA_DECAY
TRANSCRIPTIONAL_REGULATION_BY_VENTX
ATTENUATION_PHASE
DNA_DOUBLE_STRAND_BREAK_REPAIR
SUMOYLATION
REGULATION_OF_TP53_ACTIVITY_THROUGH_PHOSPHORYLATION
MITOTIC_PROPHASE
E2F_MEDIATED_REGULATION_OF_DNA_REPLICATION
TRISTETRAPROLIN_TTP_ZFP36_BINDS_AND_DESTABILIZES_MRNA
RNA_POLYMERASE_II_TRANSCRIPTION
SUMOYLATION_OF_IMMUNE_RESPONSE_PROTEINS
ACTIVATION_OF_THE_PRE_REPLICATIVE_COMPLEX
HSF1_DEPENDENT_TRANSACTIVATION
HOMOLOGY_DIRECTED_REPAIR
HDR_THROUGH_SINGLE_STRAND_ANNEALING_SSA
EXTENSION_OF_TELOMERES
SIGNALING_BY_FGFR2
0 2 4 6 8
$-\log_{10}$ p-value
