# Supplementary material for: Lactate promotes neuronal differentiation of SH-SY5Y cells by lactate-responsive gene sets through NDRG3-dependent and -independent manners
Source: J Biol Chem. 2023 May 10;299(6):104802. doi: 10.1016/j.jbc.2023.104802 (PMC10276297; doi:10.1016/j.jbc.2023.104802)
Supplement: Supplemental Figure 6 — GO term enrichment analysis of siNDRG3-treated or untreated SH-SY5Y cells by RNA-Seq.A and B, top 20 of downregulated pathways (A) and bottom 20 of upregulated pathways (B) by NDRG3 knockdown in SH-SY5Y. C, top 20 pathways which downregulated pathways downregulated by siNDRG3 transfection and upregulated by lactate treatment. [file mmc7.pdf]

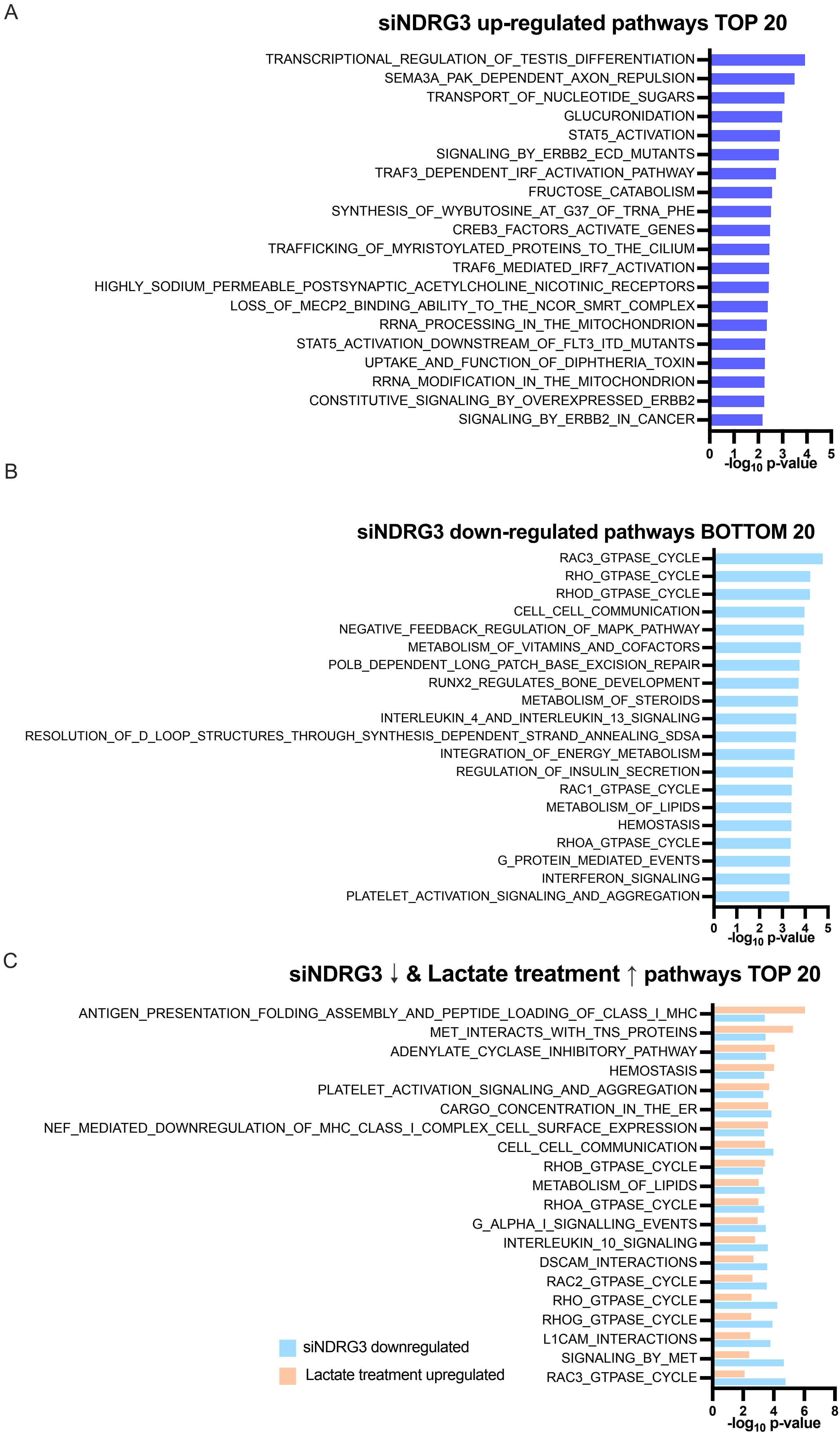
A
siNDRG3 up-regulated pathways TOP 20
TRANSCRIPTIONAL_REGULATION_OF_TESTIS_DIFFERENTIATION
SEMA3A_PAK_DEPENDENT_AXON_REPULSION
TRANSPORT_OF_NUCLEOTIDE_SUGARS
GLUCURONIDATION
STAT5_ACTIVATION
SIGNALING_BY_ERBB2_ECD_MUTANTS
TRAF3_DEPENDENT_IRF_ACTIVATION_PATHWAY
FRUCTOSE_CATABOLISM
SYNTHESIS_OF_WYBUTOSINE_AT_G37_OF_TRNA_PHE
CREB3_FACTORS_ACTIVATE_GENES
TRAFFICKING_OF_MYRISTOYLATED_PROTEINS_TO_THE_CILIUM
TRAF6_MEDIATED_IRF7_ACTIVATION
HIGHLY_SODIUM_PERMEABLE_POSTSYNAPTIC_ACETYLCHOLINE_NICOTINIC_RECEPTORS
LOSS_OF_MECP2_BINDING_ABILITY_TO_THE_NCOR_SMRT_COMPLEX
RRNA_PROCESSING_IN_THE_MITOCHONDRION
STAT5_ACTIVATION_DOWNSTREAM_OF_FLT3_ITD_MUTANTS
UPTAKE_AND_FUNCTION_OF_DIPHTHERIA_TOXIN
RRNA_MODIFICATION_IN_THE_MITOCHONDRION
CONSTITUTIVE_SIGNALING_BY_OVEREXPRESSED_ERBB2
SIGNALING_BY_ERBB2_IN_CANCER
0 1 2 3 4 5
-log10 p-value
B
siNDRG3 down-regulated pathways BOTTOM 20
RAC3_GTPASE_CYCLE
RHO_GTPASE_CYCLE
RHOD_GTPASE_CYCLE
CELL_CELL_COMMUNICATION
NEGATIVE_FEEDBACK_REGULATION_OF_MAPK_PATHWAY
METABOLISM_OF_VITAMINS_AND_COFACTORS
POLB_DEPENDENT_LONG_PATCH_BASE_EXCISION_REPAIR
RUNX2_REGULATES_BONE_DEVELOPMENT
METABOLISM_OF_STEROIDS
INTERLEUKIN_4_AND_INTERLEUKIN_13_SIGNALING
RESOLUTION_OF_D_LOOP_STRUCTURES_THROUGH_SYNTHESIS_DEPENDENT_STRAND_ANNEALING_SDSA
INTEGRATION_OF_ENERGY_METABOLISM
REGULATION_OF_INSULIN_SECRETION
RAC1_GTPASE_CYCLE
METABOLISM_OF_LIPIDS
HEMOSTASIS
RHOA_GTPASE_CYCLE
G_PROTEIN_MEDIATED_EVENTS
INTERFERON_SIGNALING
PLATELET_ACTIVATION_SIGNALING_AND_AGGREGATION
0 1 2 3 4 5
-log10 p-value
C
siNDRG3 ↓ & Lactate treatment ↑ pathways TOP 20
ANTIGEN_PRESENTATION_FOLDING_ASSEMBLY_AND_PEPTIDE_LOADING_OF_CLASS_I_MHC
MET_INTERACTS_WITH_TNS_PROTEINS
ADENYLATE_CYCLASE_INHIBITORY_PATHWAY
HEMOSTASIS
PLATELET_ACTIVATION_SIGNALING_AND_AGGREGATION
CARGO_CONCENTRATION_IN_THE_ER
NEF_MEDIATED_DOWNREGULATION_OF_MHC_CLASS_I_COMPLEX_CELL_SURFACE_EXPRESSION
CELL_CELL_COMMUNICATION
RHOB_GTPASE_CYCLE
METABOLISM_OF_LIPIDS
RHOA_GTPASE_CYCLE
G_ALPHA_I_SIGNALLING_EVENTS
INTERLEUKIN_10_SIGNALING
DSCAM_INTERACTIONS
RAC2_GTPASE_CYCLE
RHO_GTPASE_CYCLE
RHOG_GTPASE_CYCLE
L1CAM_INTERACTIONS
SIGNALING_BY_MET
RAC3_GTPASE_CYCLE
siNDRG3 downregulated
Lactate treatment upregulated
0 2 4 6 8
-log10 p-value
